# Hepatitis B virus evades the immune system by suppressing the NF-κB signaling pathway with DENND2A

**DOI:** 10.1128/spectrum.03785-23

**Published:** 2024-01-19

**Authors:** Mayuko Ide, Noriko Tabata, Yuko Yonemura, Kazuhisa Murai, Ying Wang, Atsuya Ishida, Masao Honda, Shuichi Kaneko, Satoru Ito, Hiroshi Yanagawa

**Affiliations:** 1 Research Department, Purotech Bio Inc, Yokohama, Kanagawa, Japan; 2 Department of Clinical Laboratory Medicine, Kanazawa University Graduate School of Health Medicine, Kanazawa, Ishikawa, Japan; 3 Department of Gastroenterology, Kanazawa University Graduate School of Medicine, Kanazawa, Ishikawa, Japan; University of Manitoba, Winnipeg, Manitoba, Canada

**Keywords:** hepatitis B virus, immune evasion, NF-kB, antiviral agents, drug delivery, protein-protein interactions

## Abstract

Overcoming hepatitis B virus (HBV) is a challenging problem because HBV deceives the host immune system. We have found that DENN domain-containing 2A (DENND2A) was essential for HBV maintenance, although its role remains unclear. In this study, we elucidate its function by screening a novel DENND2A-binding peptide, DENP4-3S. DENP4-3S exhibits homology to SAM and SH3 domain-containing protein 1 (SASH1), a scaffold protein involved in Toll-like receptor signaling that promotes proinflammatory cytokine production. We confirmed that DENND2A interacts with SASH1 specifically. Overexpression and knockdown experiments showed that overexpression of DENND2A suppressed the transcriptional activity of NF-κB, and the knockdown of DENND2A promoted it and the production of cytokines and interferons. Here, we constructed a fusion protein (10M-DEN3SN) consisting of an anti-asialoglycoprotein receptor antibody and DENP4-3S to deliver the peptide to hepatocytes specifically. 10M-DEN3SN inhibited the interaction between DENND2A and SASH1, and rescued SASH1 trapped by DENND2A, leading to the upregulation of NF-κB and its downstream signaling. In addition, 10M-DEN3SN suppressed HBV proliferation in PXB chimeric mice. These results with the DENND2A-binding peptide delivered into hepatocytes suggested the involvement of DENND2A, SASH, and NF-κB signaling pathway in the HBV infection and onset of hepatitis. In conclusion, this study indicates that HBV utilizes DENND2A and SASH1 to evade the immune system.

**IMPORTANCE:**

Hepatitis B virus (HBV) is a serious liver infection with no established cure, causing an abnormal host immune response. Here, we identified a novel peptide that interacts with DENN domain-containing 2A (DENND2A), a host factor essential for HBV maintenance. The resulting peptide showed sequence homology, revealing an interaction between DENND2A and the immune system regulator SASH1. This study suggests that DENND2A contributes to HBV infection by suppressing the cellular immune system by inhibiting SASH1. The DENND2A-binding peptide, incorporated into our hepatocyte-specific peptide delivery system, inhibited the DENND2A-SASH1 interaction and promoted the production of cytokines and interferons in cultured hepatocytes. As a consequence, the peptide suppressed HBV proliferation in humanized mice. We report new insights into the role of DENND2A and SASH1 in HBV maintenance and highlight the importance of the immune system.

## INTRODUCTION

Hepatitis B virus (HBV) is a major cause of chronic liver disease, such as cirrhosis and hepatocellular carcinoma. Globally, more than 250 million people are estimated to be infected with HBV (1), making it an urgent health problem. The host immune system plays an important role in the control of HBV infection (2).

We previously identified host genes that were upregulated in an HBV-persisting cell using single-cell transcriptomic analysis (3). The genes were expressed more intensely in HBV-infected cells than in uninfected cells, and the coded proteins were confirmed to colocalize with the HBV-associated protein HBc. Knockdown experiments revealed that dedicator of cytokinesis 11 (DOCK11) and DENN domain-containing 2A (DENND2A) promotes HBV replication. Thus, we considered these genes as novel targets for antiviral treatment and elucidated the role of DOCK11 for HBV replication (4). In this study, we aimed to investigate the role of DENND2A and obtain anti-HBV peptide drugs that interact with DENND2A.

DENND2A is a member of the DENN domain family that is conserved throughout evolution (5). The DENN family interacts directly with the Rab family members and functions enzymatically as Rab-specific guanine nucleotide exchange factors (GEFs). DENND2A exhibits GEF activity for Rab9 and Rab15 (6, 7), although the other roles remain unknown.

**Fig 1:**
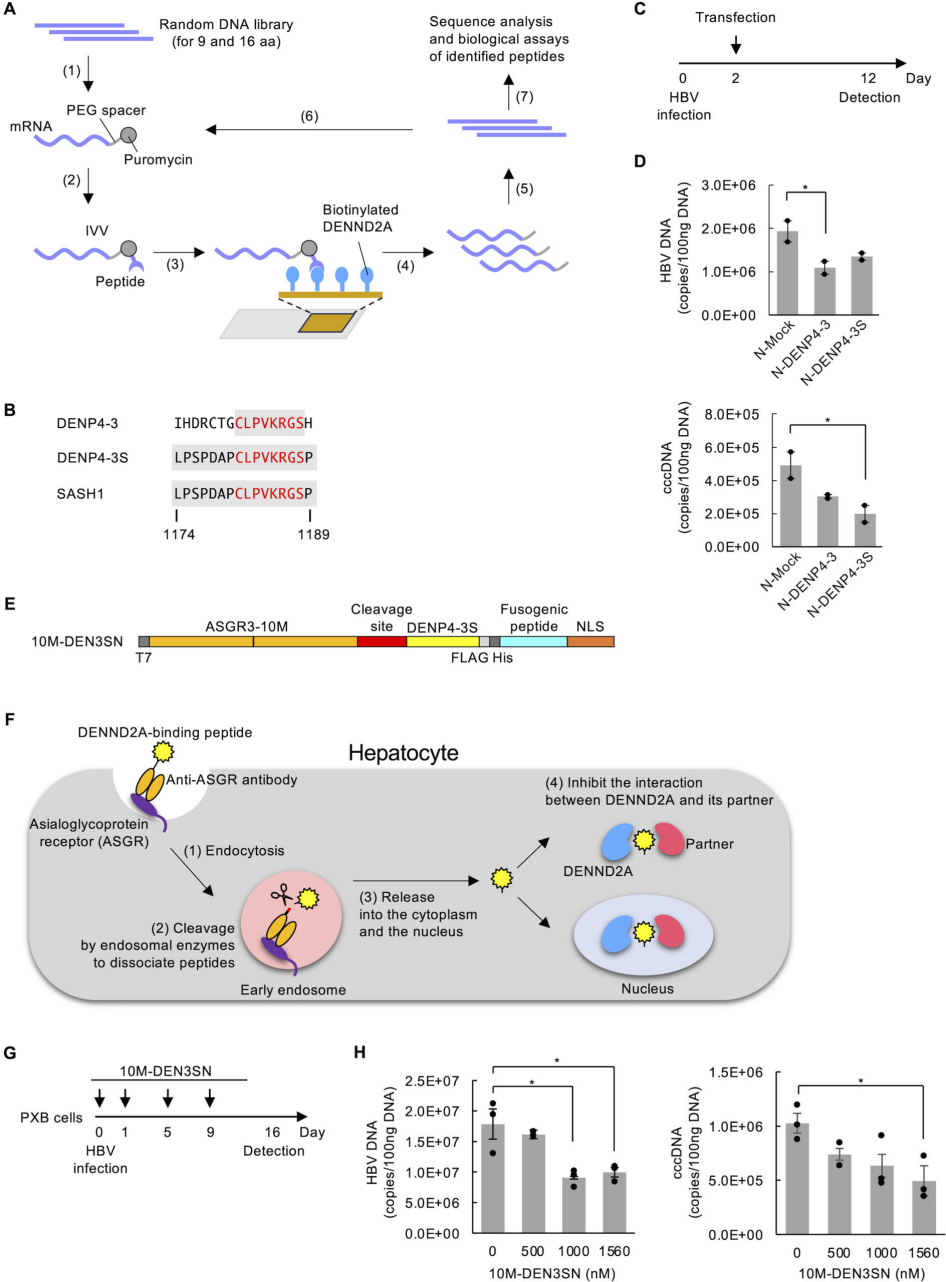
DENP4-3S was identified as a DENND2A-binding peptide with anti-HBV activity using the IVV method. (**A**) Schematic representation of the IVV method used to screen for DENND2A-binding peptide. Step 1: A random cDNA library consisting of 9 and 16 amino acid residues was transcribed and ligated with a polyethylene glycol (PEG)-puromycin spacer with a photocleavable linker. Step 2: The resulting mRNA template was translated *in vitro* to form a library of protein-mRNA conjugates (IVV molecules). Step 3: The library was injected into a microfluidic chip on which biotinylated DENND2A was immobilized, and unbound molecules were washed away. Step 4: The spacer of the bound molecules was cleaved with 365 nm UV light and eluted. Step 5: The eluted mRNA portion was amplified via RT-PCR. Step 6: The resulting DNA was used for the next round of selection. Step 7: After four selection rounds, the enriched DNA was analyzed by cloning and sequencing followed by BLAST-P analysis and biological verifications. (**B**) The amino acid sequence of the peptides identified as DENND2A-binding peptides using the IVV method and SASH1 (1174–1189 amino acids). (**C**) Schematic of the experimental design. Huh7-NTCP-YFP cells were infected with HBV 2 days before transfection and maintained for 10 days after transfection. (**D**) qPCR analyses of HBV DNA and covalently closed circular DNA (cccDNA) levels in Huh7-NTCP-YFP cells transfected with a plasmid encoding nuclear localization signal (NLS)-tagged mock (N-Mock), DENP4-3 (N-DENP4-3), or DENP4-3S (N-DENP4-3S). Data are presented as the mean ± SD pooled from two independent experiments. *P < 0.05. (**E**) Design of 10M-DEN3SN, a fusion between DENND2A-binding peptide (DENP4-3S) and anti-ASGR scFv antibody (ASGR3-10M). A cleavage site for an endosomatic enzyme, fusogenic peptide for release from early endosomes, and NLS for nuclear localization are also present in the fusion protein. (**F**) Proposed mechanism of action of 10M-DEN3SN in hepatocytes. Step 1: 10M-DEN3SN is endocytosed into early endosomes via anti-ASGR antibody. Step 2: 10M-DEN3SN is cleaved at the cleavage site by the enzyme furin in endosomes. Step 3: The peptide DENP4-3S is released into the cytoplasm via the fusogenic peptide and enters into the nucleus via NLS. Step 4: DENP4-3S inhibits the interaction between DENND2A and its partner proteins in the cytoplasm and nucleus. (**G**) Schematic of the experimental design. PXB cells were treated with 10M-DEN3SN at 1, 5, and 9 days after HBV infection. (**H**) qPCR analyses of HBV DNA and cccDNA in PXB cells treated with 10M-DEN3SN at 16 days after infection. Data are presented as the mean ± SD pooled from three independent experiments. *P < 0.05.

We have developed the *in vitro* virus (IVV) method for studying protein-protein interactions (PPIs) (8–10) and evolutionary protein engineering (11–13). The method is an mRNA display for construction of a library of protein-mRNA conjugates, linking each protein to its encoding mRNA (8) (Fig. 1A). The IVV method enables screening of the proteins [including peptides (14–16) and antibodies (11, 17)] with high affinity for the target. Subsequent verification of their effects on the target’s PPI and biological function achieves biologically functional outcomes. Here, we performed the IVV selection for novel DENND2A-binding peptides. In consequence, we identified DENP4-3S, which showed homology to the sequences of SAM and SH3 domain-containing protein 1 (SASH1). This DENND2A-binding peptide resembling SASH1 also demonstrated antiviral activity against HBV. To deliver the peptide into hepatocytes, we subsequently fused DENP4-3S with an antibody against an asialoglycoprotein receptor (ASGR) which was also identified by the IVV method (4). We confirmed that the fusion protein named 10M-DEN3SN rescued SASH1 from the inhibition by DENND2A in the Toll-like receptor 4 (TLR4) signaling. It leads to the production of cytokines and interferons (IFNs) in response to the virus infection. This mechanism of action of 10M-DEN3SN explains the role of DENND2A and SASH1 in HBV infection.

## RESULTS

### *In vitro* selection of DENND2A-binding peptide by the IVV method

To obtain the DENND2A-binding peptides, we utilized IVV selection as illustrated schematically in Fig. 1A. There are two isoforms of DENND2A. We used isoform 2, consisting of the u-DENN domain and DENN domain, as a bait for the selection. Four rounds of selection resulted in 22 clones, and 15 clones of them were duplicates of the same sequence, DENP4-3 (Fig. 1B). The numerous duplicated sequences obtained suggest that optimal sequences that strongly bind to DENND2A have been selected. DENP4-3 has homology to 1174–1189 amino acids of SASH1 (UniProtKB-O94885 SASH1_HUMAN) (Fig. 1B). This sequence is located in the proline-rich domain (1141–1222 amino acids), suggesting the possible interaction between DENND2A and SASH1. Considering the importance of DENND2A in HBV maintenance, disruption of DENND2A-SASH1 interaction and its function may inhibit HBV infection. Then, we synthesized a partial sequence of SASH1 to which DENP4-3 has homology and named it DENP4-3S. When the nuclear localization signal (NLS)-tagged DENP4-3 or DENP4-3S (N-DENP4-3, N-DENP4-3S) was transfected into Huh7-NTCP-YFP cells infected with HBV (Fig. 1C), the levels of HBV DNA and covalently closed circular DNA (cccDNA) in the cells were suppressed (Fig. 1D).

Next, we constructed the fusion protein 10M-DEN3SN by incorporating DENP4-3S into the hepatocyte-specific peptide delivery system we developed (4) (Fig. 1E; Table S1). A schematic mechanism of action of 10M-DEN3SN is shown in Fig. 1F. The fusion protein is endocytosed to early endosomes by the anti-ASGR antibody, ASGR3-10M. ASGR3-10M is a single-chain antibody identified by the IVV method with the extracellular domain of ASGR1 and ASGR2 as a bait (4) and confirmed to interact with ASGR and internalize into hepatocytes. The endocytosed protein was cleaved by furin protease in the endosome, resulting in peptide dissociation. The peptide is released into the cytoplasm by fusogenic peptide S28 and translocated to the nucleus via NLS. Thus, the interaction between DENND2A and its partner proteins is inhibited by the DENND2A-binding peptide in the cytoplasm and the nucleus. We have demonstrated that this hepatocyte-specific peptide delivery system works as shown in Fig. 1F using a DOCK11-binding peptide (4), consisting of 16 residues as well as DENND2A-binding peptide.

We verified in the same way for the DENND2A-binding peptide. 10M-DEN3SN was cleaved by furin at 37°C for 1 hour, and the cleaved N-terminal and C-terminal regions were detected with the expected molecular weight (Fig. S1A and B). Huh7 cells were treated with 10M-GFP-DEN3SN (10M-DEN3SN specially containing GFP at the C-terminal region) for 5 hours and then fractionated to membrane, cytosol, and nuclear fractions. The whole construct was observed in the membrane and cytosol fraction, and cleaved C-terminal region was observed in the cytosol and nuclear fraction (Fig. S1C), suggesting that 10M-DEN3SN attached to the cell membrane and was cleaved in the cytoplasm after internalization, and only the cleaved C-terminal region localized to the nucleus. These results indicate that the cleavage occurs *in vitro* and *in vivo,* and the drug delivery system containing DENND2A-binding peptide works as expected.

Finally, we investigated whether 10M-DEN3SN has an inhibitory effect on HBV proliferation in primary human hepatocytes, PXB cells. PXB cells were treated with 10M-DEN3SN followed by a measurement of HBV DNA and cccDNA levels (Fig. 1G). As a result, 10M-DEN3SN decreased HBV proliferation in a concentration-dependent manner (Fig. 1H). These results confirmed that 10M-DEN3SN has anti-HBV activity in hepatocytes.

### 10M-DEN3SN inhibits the interaction between DENND2A and SASH1

**Fig 2:**
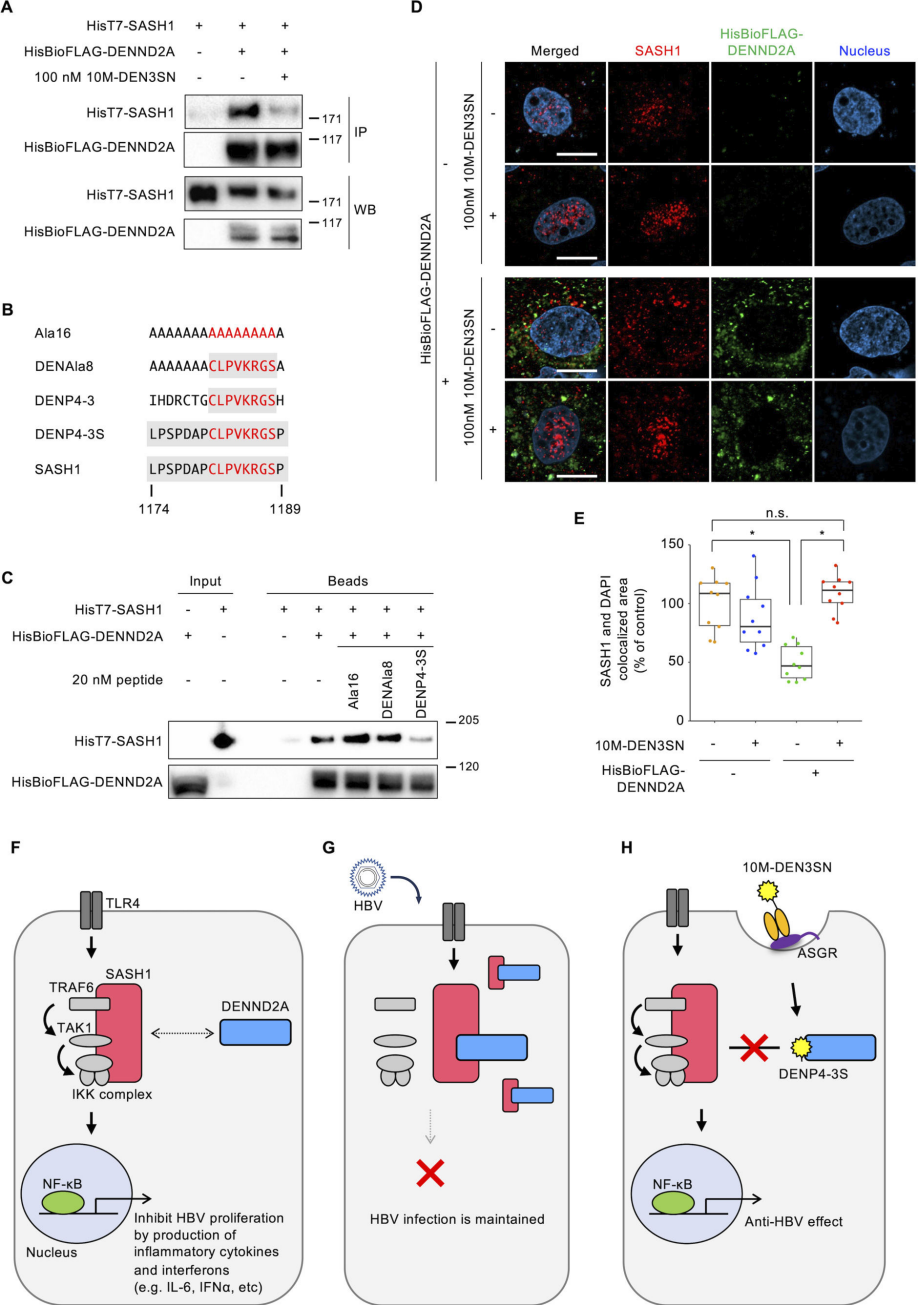
DENP4-3S inhibited the interaction between DENND2A and SASH1. (**A**) Coimmunoprecipitation assay using HisT7-SASH1 and HisBioFLAG-DENND2A coexpressed in HEK293T cells treated with 10M-DEN3SN for 24 hours. The lysates were immunoprecipitated (IP) by streptavidin and western blotted (WB) using anti-T7 or anti-FLAG-tag antibody. (**B**) The amino acid sequence of the peptides identified as DENND2A-binding peptides using the IVV method and SASH1 (1174–1189 amino acids). (**C**) Pulldown assays in the presence of 20 nM peptides using HisT7-SASH1 and HisBioFLAG-DENND2A. HEK293T cells were transfected with a plasmid encoding HisT7-SASH1, and the whole-cell lysates were used for pulldown assays with HisBioFLAG-DENND2A-immobilized beads in the presence of each peptide. Each fraction was separated by 3%–8% SDS-PAGE and then analyzed by western blotting with an antibody against the T7-tag or FLAG-tag. (**D, E**) Huh7 cells were transfected with HisBioFLAG-tag or HisBioFLAG-DENND2A for 24 hours and then treated with or without 100 nM 10M-DEN3SN for 5 hours. Immunofluorescence staining using anti-FLAG-tag (AlexaFluor488, green) and anti-SASH1 (AlexaFluor647, red) was then performed. DAPI was used to stain nuclei (blue). The samples were observed using confocal microscopy. Scale bars, 10 µm. (**E**) The ratio of area where SASH1 and DAPI colocalized was quantified. Boxplots: center line, median; box limits, 25th to 75th percentiles; whiskers, minimum to maximum. Data are presented as the mean ± SEM pooled from 10 independent experiments. (**F–H**) Conceptual schematic representation of the mechanism of action of 10M-DEN3SN in a hepatocyte. (**F**) SASH1 is a scaffold protein in TLR4 signaling pathway that activates NF-κB and then increases the production of proinflammatory cytokines and interferons. DENND2A interacts with SASH1 and modulates its activity in response to the immune system. (**G**) HBV infection induces an increase in the expression of DENND2A. DENND2A promotes HBV proliferation by trapping SASH1 and inhibiting its downstream signaling. (**H**) 10M-DEN3SN endocytosed into hepatocyte releases DENND2A-binding peptide DENP4-3S. DENP4-3S inhibits the interaction between DENND2A and SASH1, resulting in the rescue of the TLR4 signaling pathway and immune system with anti-HBV activity.

To investigate the mechanism of action of 10M-DEN3SN, we focused on protein-protein interactions. First, we performed a coimmunoprecipitation assay to examine whether DENND2A interacts with SASH1 and whether 10M-DEN3SN inhibits the interaction in the cells. The cells transfected with FLAG-tagged biotinylated DENND2A and HisT7-tagged SASH1 were treated with 10M-DEN3SN, and then the lysate was purified with streptavidin beads. The results showed that SASH1 specifically bound to DENND2A in cells and that the interaction was inhibited by 10M-DEN3SN (Fig. 2A). We additionally examined whether DENP4-3S inhibits the interaction between DENND2A and SASH1 *in vitro*. DENND2A was immobilized on beads and examined for binding of HisT7-tagged SASH1 in the presence of DENP4-3S. Peptides with all or part of DENP4-3S replaced with alanine, Ala16 and DENAla8, were used as negative controls (Fig. 2B). Ala16 consists of all 16 residues being replaced with alanine. DENAla8 is a peptide with eight residues of DENP4-3S identified to be homologous to SASH1 by IVV screening, and the remaining eight residues are replaced with alanine. As a result, DENP4-3S inhibited the interaction between DENND2A and SASH1 (Fig. 2C). Ala16 did not inhibit the binding at all, but the inhibition became stronger as the peptide sequence was replaced by that of SASH1, suggesting the effectiveness of DENP4-3S in blocking the interaction of SASH1 and DENND2A. The negative control peptides were confirmed to have no anti-HBV effect by overexpressing in PXB cells (Fig. S2).

We monitored the effect of DENND2A and 10M-DEN3SN on SASH1 localization. Huh7 cells were transfected with HisBioFLAG-tag (control) or HisBioFLAG-DENND2A and then treated with 10M-DEN3SN for 5 hours (Fig. 2D and E). Endogenous SASH1 was mainly localized to the nucleus in the control cells, while in the DENND2A-transfected cells, it was reduced by 50% and shifted to the cytoplasm. The localization change was inhibited by 10M-DEN3SN and SASH1 returned to the nucleus. In this assay, overexpressed DENND2A was localized to the cytoplasm, as well as endogenous DENND2A (Fig. S3).

SASH1 is reported to be a scaffold protein in TLR4 signaling that activates NF-κB and MAPKs, resulting in increased production of cytokines and interferons (Fig. 2F) (18). SASH1 transmits the signal to downstream by binding to TRAF6, TAK, and the IκB kinase (IKK) complex independently. TLRs activate intracellular antiviral mechanisms and the production of antiviral effectors, such as IFNs and proinflammatory cytokines. Cytokines, such as interleukin-6 (IL-6) and IL-1β, have been reported to inhibit HBV replication and transcription (19, 20). IFNα and IFNβ suppress HBV infection *in vitro* and *in vivo*; thus, these interferons have been used to treat HBV (21, 22). Since the above experiments confirmed the specific interaction between DENND2A and SASH1, it is conceivable that DENND2A interacts with SASH1 and modulates its activity in response to the immune system. HBV avoids the host immune system by suppressing the expression and activation of TLRs and cellular signaling pathways (23–28). DENND2A is essential for HBV proliferation, and its expression is upregulated in HBV-infected cells (3). Therefore, we considered that DENND2A may contribute to HBV proliferation by trapping SASH1 and inhibiting the TLR4 signaling pathway and its downstream production of cytokines and interferons (Fig. 2G). Based on this hypothesis, 10M-DEN3SN may rescue SASH1 and restore the cellular immune system by inhibiting DENND2A (Fig. 2H).

### 10M-DEN3SN promotes TLR4 signaling

**Fig 3:**
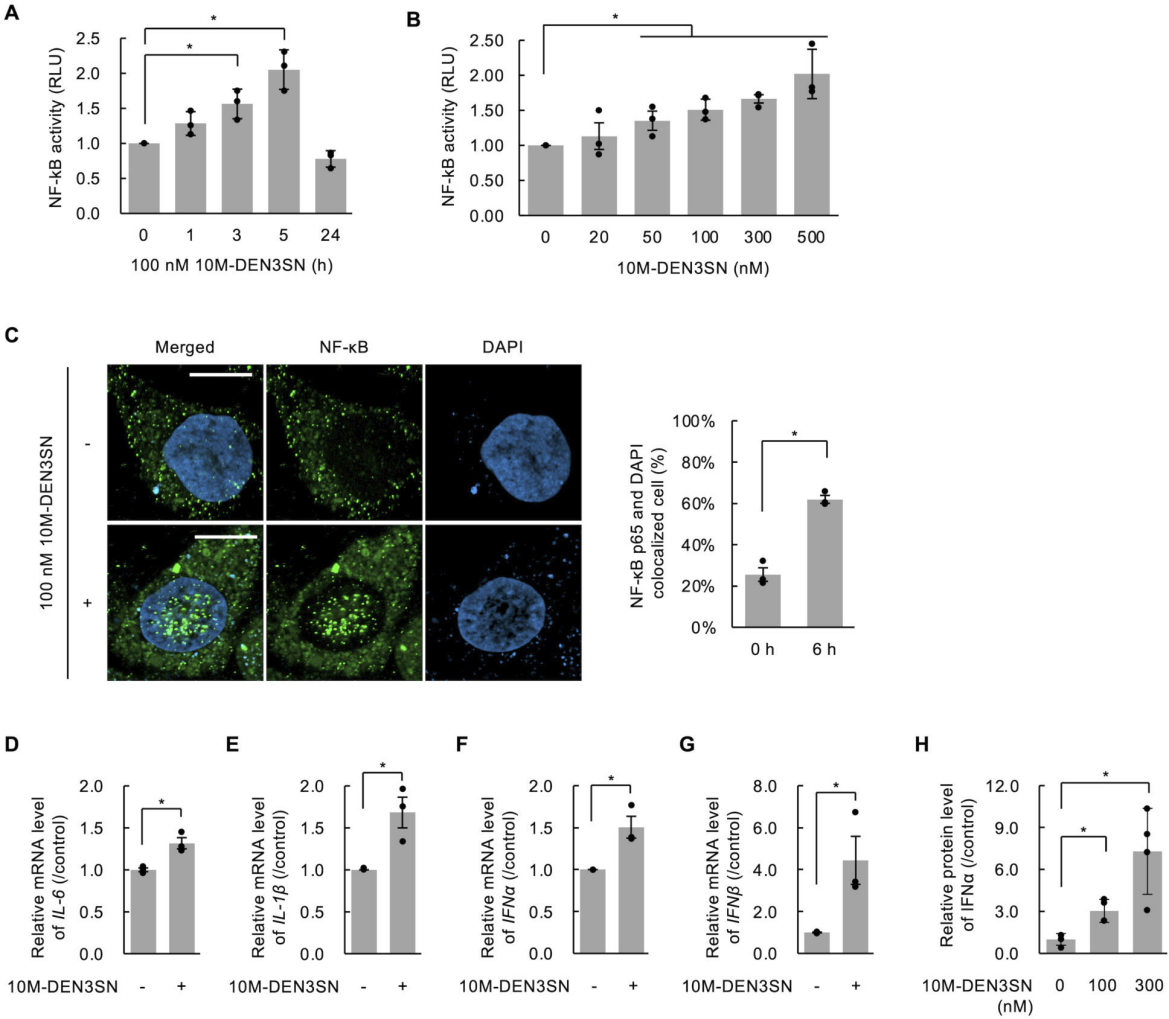
10M-DEN3SN promoted the NF-κB signaling pathway. (**A, B**) HepG2-NFκB-Luc cells were treated with 100 nM 10M-DEN3SN for 0–24 hours (**A**) or with 0–100 nM 10M-DEN3SN for 5 hours (**B**). NF-κB transcriptional activity was measured by luciferase assay. The relative luciferase unit (RLU) was normalized by comparing it to the control. (**C**) Immunofluorescence staining of Huh7 cells using anti-NF-κB antibody (AlexaFluor488, green) after treatment with 0 or 100 nM 10M-DEN3SN for 5 hours. The samples were observed using confocal microscopy. Scale bars, 10 µm. Nuclear NF-κB localization was scored for greater than 100 cells. The ratio under the indicated conditions is shown. (**D–G**) Huh7 cells were treated with 100 nM 10M-DEN3SN for 5 hours, and IL-6 (**D**), IL-1β (**E**), IFNα (**F**), and IFNβ (**G**) mRNA were detected by qRT-PCR. (**H**) Huh7 cells were treated with 0–300 nM 10M-DEN3SN for 5 hours, and the medium was analyzed by ELISA targeting IFNα. Error bars represent ±SD. *P < 0.05.

To investigate whether 10M-DEN3SN affects the activity of the NF-κB transcription factor, a luciferase reporter gene assay was performed in HepG2 cells transfected with the pTA-NFκB-luciferase reporter vector (HepG2-NFκB-Luc cells). HepG2-NFκB-Luc cells were treated with 100 nM 10M-DEN3SN for 0–24 hours, and then the lysates were used for reporter gene assays. The results showed that NF-κB transcriptional activity was doubled after 5 hours of treatment with 10M-DEN3SN (Fig. 3A). The NF-κB transcriptional activity was also induced in a concentration-dependent manner after 5 hours of treatment with 0–500 nM 10 M-DEN3SN (Fig. 3B). Given that NF-κB acts as a transcription factor upon translocation from the cytoplasm to the nucleus, we performed an immunofluorescence assay using an anti-NF-κB antibody. As a result, 10M-DEN3SN induced the nuclear localization of NF-κB (Fig. 3C). Furthermore, we analyzed the transcription of cytokines regulated by NF-κB in the cells treated with or without 100 nM 10M-DEN3SN for 5 hours. Quantitative RT-PCR analyses showed that in the cells treated with 10M-DEN3SN, IL-6, IL-1β, IFNα, and IFNβ mRNA levels were increased (Fig. 3D through G). The effect of increased mRNA levels on these cytokines and interferons was also observed in HepG2-NFκB-Luc cells (Fig. S4). We also assessed the protein level of IFNα in the medium after treatment with 0–300 nM 10M-DEN3SN for 5 hours by ELISA. As a result, IFNα protein was increased in the concentration-dependent manner of 10M-DEN3SN (Fig. 3H).

The major ligand for the TLR4 signaling pathway is LPS (lipopolysaccharide), a component of gram-negative bacteria. 10M-DEN3SN used in this study is LPS free because it was expressed in cultured eukaryotic cells, not in bacteria. In addition, 10M-DEN3SN did not affect the mRNA and protein levels of DENND2A and SASH1 (Fig. S5). These results suggest that 10M-DEN3SN promoted the transcriptional activity of NF-κB by affecting the function of DENND2A or SASH1.

### 10M-DEN3SN recovers NF-κB transcriptional activity suppressed by DENND2A

**Fig 4:**
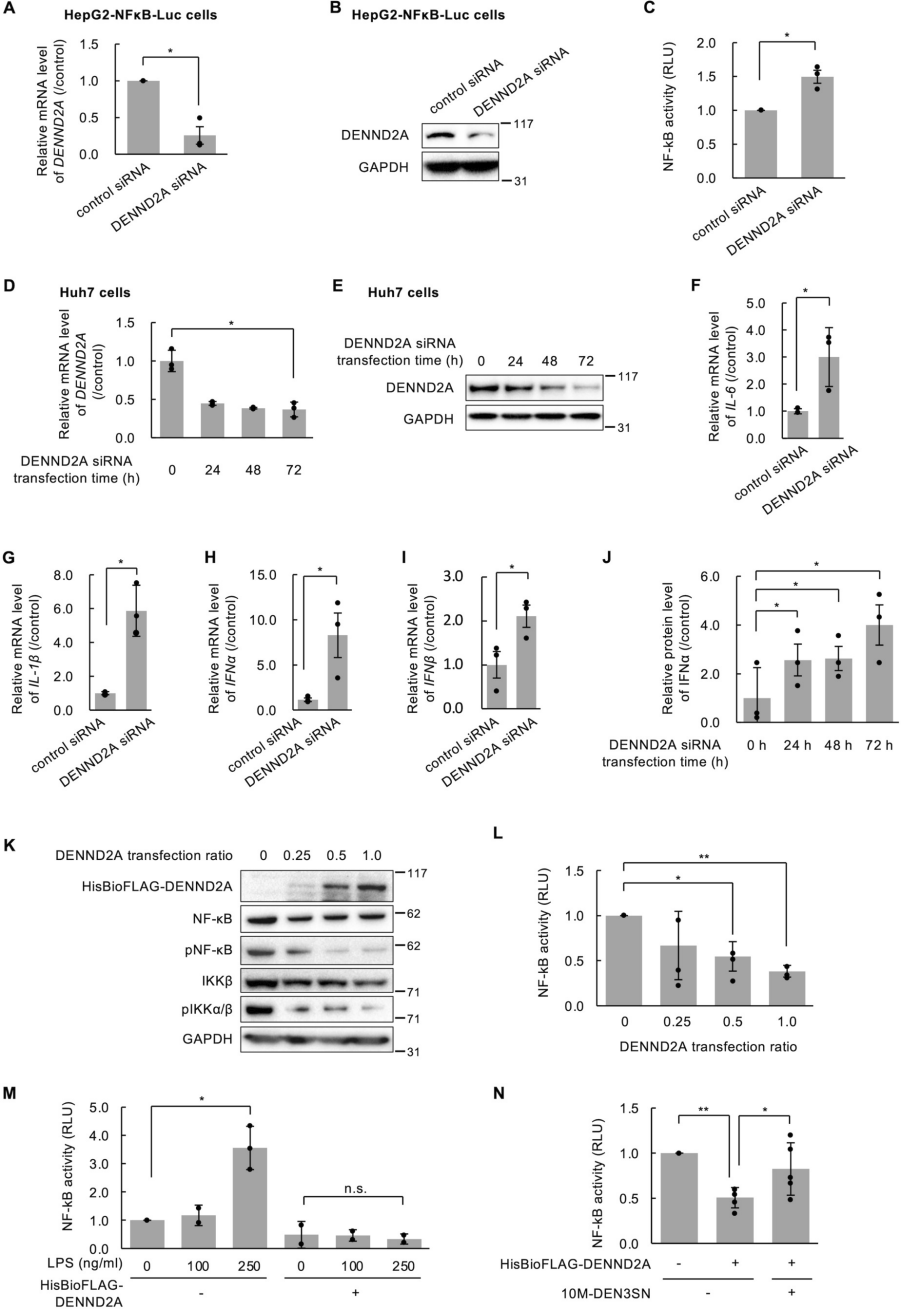
10M-DEN3SN rescued the NF-κB signaling pathway suppressed by DENND2A. (**A–C**) HepG2-NFκB-Luc cells were transfected with DENND2A or control siRNA for 72 hours. DENND2A mRNA was detected by qRT-PCR (**A**), and DENND2A protein was detected by western blotting (**B**). GAPDH is shown to verify equal loading. NF-κB transcriptional activity was measured by luciferase assay (**C**). The RLU was normalized by comparing it to the control. (**D–J**) Huh7 cells were transfected with DENND2A siRNA for 24, 48, and 72 hours and control siRNA for 72 hours (as transfection of DENND2A siRNA for 0 hours). DENND2A mRNA was detected by qRT-PCR (**C**), and DENND2A protein was detected by western blotting (**E**). IL-6 (**F**), IL-1β (**G**), IFNα (**H**), and IFNβ (**I**) mRNA in cells transfected for 72 hours were detected by qRT-PCR. The protein level of IFNα in the medium was analyzed by ELISA (**J**). (**K, L**) HepG2-NFκB-Luc cells were transfected with HisBioFLAG-DENND2A at 0, 0.25, 0.5, and 1-fold of the specified amount. After 24 hours, the expression or phosphorylation levels of NF-κB and IKKβ in whole-cell lysates were analyzed by western blotting using antibodies against NF-κB, pNF-κB, IKKβ, and pIKKα/β (**K**), and luciferase activity was measured by luciferase assay (**L**). (**M, N**) HepG2-NFκB-Luc cells were transfected with HisBIoFLAG-DENND2A for 24 hours. The cells were treated with 0–250 ng/mL LPS (**M**) or 0–100 nM 10M-DEN3SN (**N**) for 5 hours, and then luciferase activity was measured. Error bars represent ±SD. *P < 0.05; **P < 0.005; n.s., not significant.

Next, we examined whether DENND2A affects the transcriptional activity of NF-κB. HepG2-NFκB-Luc cells were transfected with DENND2A-specific siRNA (DENND2A siRNA) or nontargeting siRNA (control siRNA). The knockdown of endogenous DENND2A was confirmed by quantitative RT-PCR and western blotting (Fig. 4A and B). The reporter gene assay showed that NF-κB luciferase reporter activity was increased in cells transfected with DENND2A siRNA relative to control cells (Fig. 4C). We also examined the effect of DENND2A knockdown on downstream of NF-κB transcriptional activity. Huh7 cells were transfected with DENND2A siRNA or control siRNA, and then the suppression level of DENND2A was confirmed (Fig. 4D and E). DENND2A knockdown in Huh7 cells led to elevated mRNA levels of IL-6, IL-1β, IFNα, and IFNβ (Fig. 4F through I). ELISA assay showed that the IFNα concentration in the medium was increased in the DENND2A knockdown time-dependent manner (Fig. 4J). Since DENND2A was suppressed in the knockdown time-dependent manner in the mRNA and protein level (Fig. 4D and E), it is suggested that the expression levels of DENND2A and IFNα are inversely proportional.

We further examined the effect of DENND2A overexpression on the transcriptional activity of NF-κB. When HepG2-NFκB-Luc cells were transfected with the DENND2A expression vector at 0, 0.25, 0.5, and 1-fold of the specified amount, DENND2A gradually expressed depending on the vector amount, while NF-κB and IKKα/β phosphorylation were decreased inversely (Fig. 4K). We performed the reporter gene assay with HepG2-NFκB-Luc cells transfected with the specified amount of DENND2A expression vector. The result showed that the reporter activity decreased in response to increased DENND2A expression (Fig. 4L). To investigate the pathway in which DENND2A affects NF-κB transcriptional activity, HepG2-NFκB-Luc cells overexpressing DENND2A were then treated with 0–250 ng/mL LPS. The reporter gene assay showed that NF-κB transcriptional activity was promoted in an LPS concentration-dependent manner, although overexpressioned DENND2A inhibited it (Fig. 4M). We also performed the reporter gene assay with DENND2A-transfected cells treated with 10M-DEN3SN instead of LPS. As a result, the suppression of NF-κB transcriptional activity by DENND2A was restored by 10M-DEN3SN (Fig. 4N). These results suggest that 10M-DEN3SN inhibits DENND2A in TLR4 signaling pathway, resulting in the recovery of NF-κB transcriptional activity.

### 10M-DEN3SN requires SASH1 to promote NF-κB transcriptional activity

**Fig 5:**
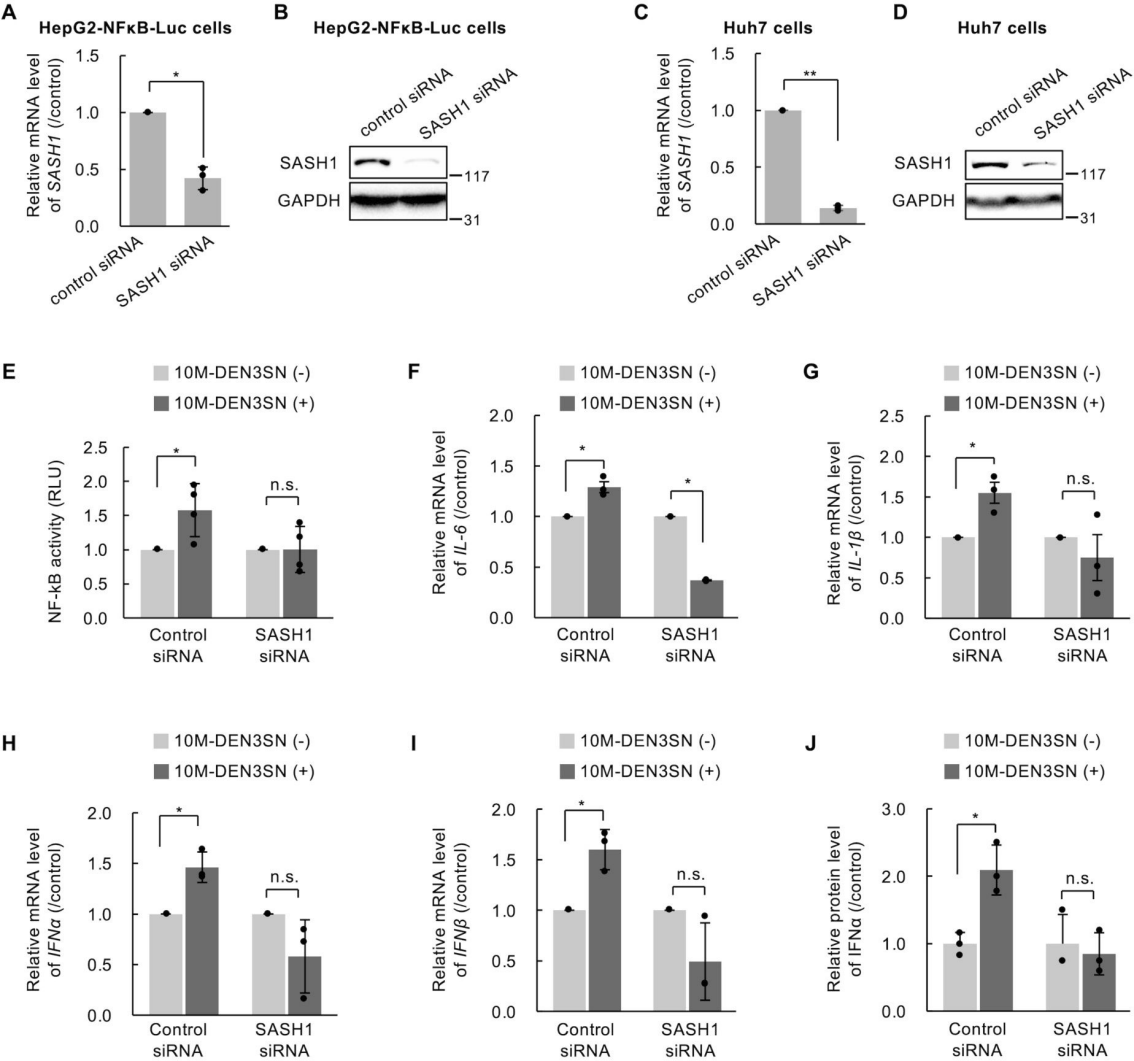
10M-DEN3SN promoted NF-κB signaling pathway activation via SASH1. (**A, B**) HepG2-NFκB-Luc cells were transfected with SASH1 or control siRNA for 72 hours. SASH1 mRNA was detected by qRT-PCR (**A**), and SASH1 protein was detected by western blotting (**B**). GAPDH is shown to verify equal loading. (**C, D**) Huh7 cells were transfected with SASH1 or control siRNA for 72 hours. SASH1 mRNA was detected by qRT-PCR (**C**), and SASH1 protein was detected by western blotting (**D**). (**E**) HepG2-NFκB-Luc cells were transfected with SASH1 or control siRNA for 72 hours and then treated with 0 or 100 nM 10M-DEN3SN for 5 hours. NF-κB transcriptional activity was measured by luciferase assay. The RLU was normalized by comparing it to the control. (**F–J**) Huh7 cells were transfected with SASH1 or control siRNA for 72 hours and then treated with 0 or 100 nM 10M-DEN3SN for 5 hours. IL-6 (**F**), IL-1β (**G**), IFNα (**H**), and IFNβ (**I**) mRNA levels were detected by qRT-PCR. The protein level of IFNα in the medium was analyzed by ELISA (**J**). Error bars represent ±SD. *P < 0.05; **P < 0.005; n.s., not significant.

It has been reported that knockdown of SASH1 decreases NF-κB luciferase reporter activity and IL-6 production, whereas overexpression of SASH1 increases both in endothelial cells (18). We examined whether SASH1 also promotes the NF-κB signaling pathway in hepatocytes. HepG2-NFκB-Luc cells and Huh7 cells were transfected with SASH1-specific siRNA (SASH1 siRNA) or control siRNA and then treated with LPS followed by the reporter gene assay and quantitative RT-PCR. The effect of the knockdown of endogenous SASH1 was confirmed in both mRNA and protein levels (Fig. 5A through D). NF-κB luciferase reporter activity was promoted with LPS treatment, although the promotion was suppressed by SASH1 knockdown in HepG2-NFκB-Luc cells (Fig. S6A). Similarly, the knockdown of endogenous SASH1 in Huh7 cells also suppressed the LPS-induced increase of IL-6, IL-1β, IFNα, and IFNβ mRNA levels (Fig. S6B through E). These results confirmed that SASH1 promotes NF-κB transcriptional activity and the production of cytokines and interferons in cultured hepatocytes.

Then, we examined whether SASH1 contributes to the promotion of NF-κB transcriptional activity by 10M-DEN3SN. HepG2-NFκB-Luc cells were transfected with SASH1 or control siRNA and then treated with 100 nM 10M-DEN3SN for 5 hours. The results of the luciferase assay showed that NF-κB transcriptional activity was not affected by 10M-DEN3SN in SASH1 knockdown cells (Fig. 5E), although it was promoted by 10M-DEN3SN in control cells as confirmed in Fig. 3. The increase of the cytokine and interferon mRNA induced by 10M-DEN3SN was suppressed by the SASH1 knockdown (Fig. 5F through I). 10M-DEN3SN also increased IFNα protein level in the medium of control cells, while this facilitative effect was inhibited by SASH1 knockdown (Fig. 5J). These results suggest that SASH1 is required for 10M-DEN3SN to promote the transcriptional activity of NF-κB.

### 10M-DEN3SN suppresses HBV infection *in vivo*

**Fig 6:**
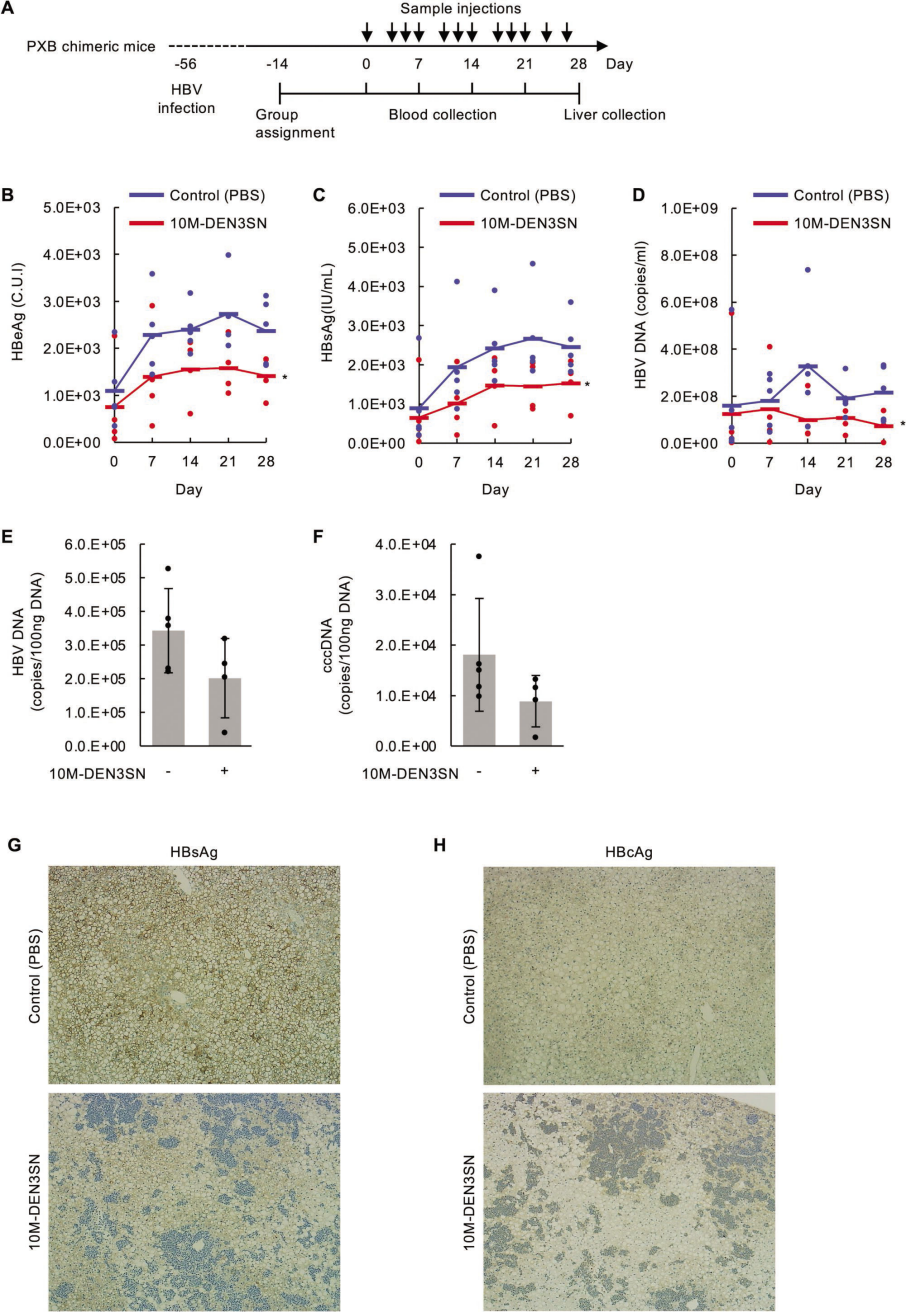
10M-DEN3SN inhibited HBV proliferation in PXB chimeric mice. (**A**) Schematic of the experimental design. (**B–D**) Hepatitis B e antigen (HBeAg) (**B**), hepatitis B surface antigen (HBsAg) (**C**), and HBV DNA (**D**) levels in the blood for 0–28 days. (**E, F**) HBV DNA (**E**) and cccDNA (**F**) in the liver collected on day 28. Error bars represent ±SD. (**G, H**) Histopathologic examination of immunostaining for HBsAg (**G**) and hepatitis B core antigen (HBcAg) (**H**) in liver tissue collected on day 28. Hepatocytes positive for antigen were detected as brown staining. Staining intensity was classified into four levels: +++, distinct and extensive to full; ++, faint to distinct and extensive to full; +, faint and partial; −, negative. HBsAg staining intensity was +++ in liver tissue treated without 10M-DEN3SN, whereas ++ in those treated with 10M-DEN3SN (**G**). HBcAg staining intensity was ++ in liver tissue treated without 10M-DEN3SN, whereas + in those treated with 10M-DEN3SN (**H**).

To further confirm the anti-HBV effect of 10M-DEN3SN, we administered 10M-DEN3SN to PXB chimeric mice. PXB chimeric mice transplanted with human hepatocytes were infected with HBV 56 days before the assay. The mice were intraperitoneally administered with 10M-DEN3SN every 2–3 days after assay initiation (Fig. 6A). Then, we collected blood after 7, 14, 21, and 28 days and livers after 28 days. One of the five 10M-DEN3SN-treated mouse died during the course of the study due to a case that often occurs in SCID mouse strains, so the remaining four were analyzed. The statistical values are shown in Table S5. The human albumin in blood remained sufficient throughout the assay, confirming that this assay system provided results in human liver cells. The necropsy showed no inflammation in the liver or other internal organs, suggesting that 10M-DEN3SN had no antigenicity. The body weight of control-treated mice was reduced by 2%–10% and that of 10M-DEN3SN-treated mice was reduced by 6%–16% (Fig. S7A). In addition, the measurement of ALT showed that 10M-DEN3SN did not make hepatitis worse compared to the control (Fig. S7B). These results indicate that 10M-DEN3SN induces less damage to the liver.

The results of monitoring hepatitis B e antigen (HBeAg), hepatitis B surface antigen (HBsAg), and HBV DNA levels in the blood showed that the administration of 10M-DEN3SN decreased these concentrations during the entire assay period (Fig. 6B through D). After the entire assay period, HBV DNA and cccDNA levels in the liver were detected by RT-PCR, and HBsAg and hepatitis B core antigen (HBcAg) levels were detected by histological examination of liver sections. The results showed that HBV DNA (Fig. 6E) and cccDNA (Fig. 6F) levels in the liver tended to decrease with the administration of 10M-DEN3SN. Staining for HBsAg (Fig. 6G) and HBcAg (Fig. 6H) in the liver sections showed that the liver tissue in the control group was uniformly stained brown, while the staining was reduced in the chimeric mice treated with 10M-DEN3SN. These results indicate that 10M-DEN3SN inhibited the HBV proliferation in the liver of chimeric mice, resulting in decreased HBsAg and HBcAg.

### Treatment of HBV with 10M-DEN3SN in combination with nucleoside analog

**Fig 7:**
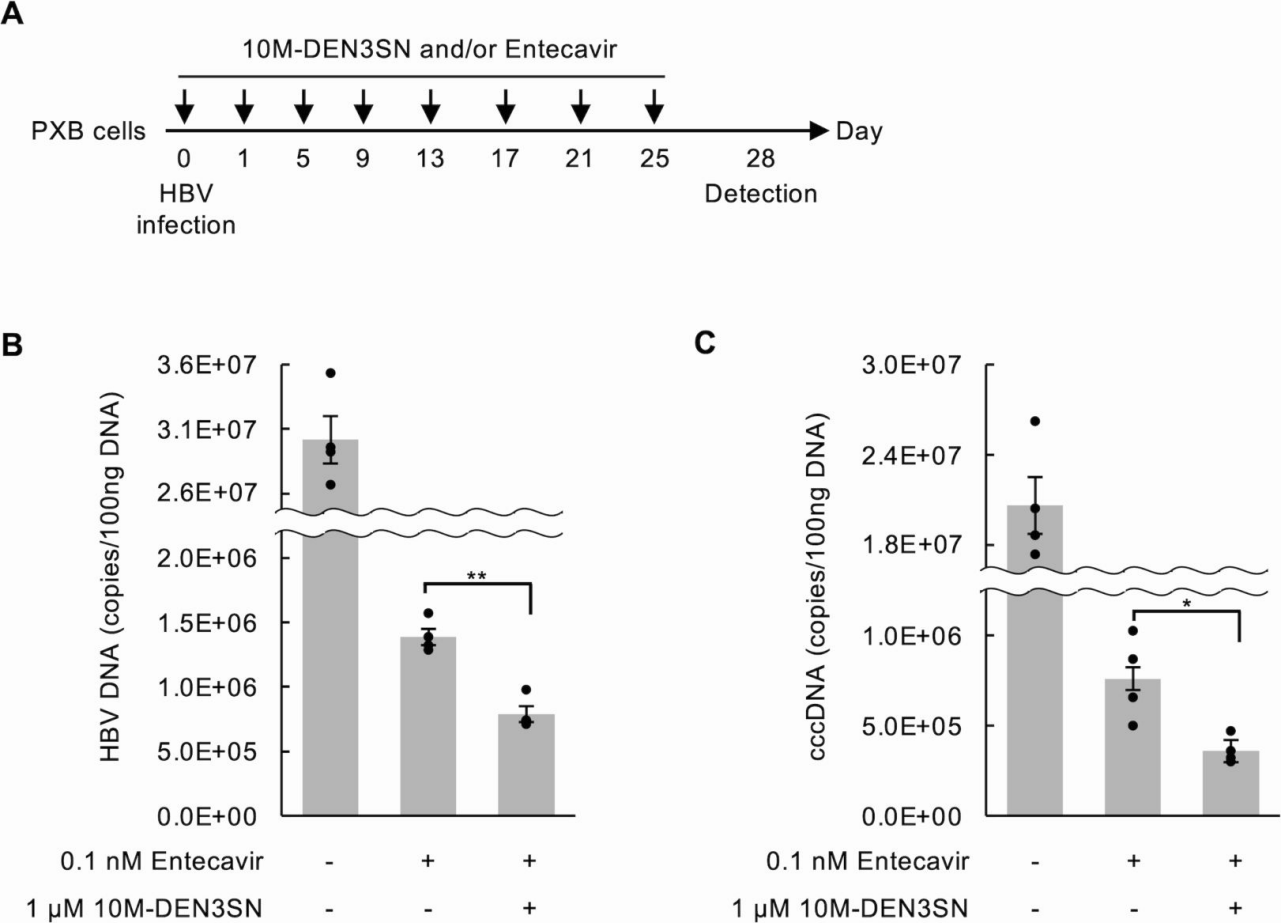
Combination effect of 10M-DEN3SN and entecavir. (**A**) Schematic of the experimental design. (**B, C**) qPCR analyses of HBV DNA (**B**) and cccDNA (**C**) in PXB cells treated with or without 0.1 nM entecavir in the absence or presence of 1 µM 10M-DEN3SN. Error bars represent ±SD. *P < 0.05; **P < 0.005.

Currently, nucleoside analogs represented by entecavir (29) are used for the treatment of HBV, and their suppressive effect has been confirmed. Therefore, we investigated the combined effect of entecavir and 10M-DEN3SN. PXB cells infected with HBV were continuously treated with 10M-DEN3SN and entecavir or each alone for 25 days, and the amount of HBV DNA and cccDNA in the cells was examined on day 28 (Fig. 7A). The results showed that both DNA levels were more remarkably reduced in the cells treated with 10M-DEN3SN together with entecavir compared to those treated with entecavir alone (Fig. 7B and C).

## DISCUSSION

The immune response of host cells plays an important role in HBV chronic infection (30). HBV particles and antigens inhibit TLR-induced antiviral activity, such as the NF-κB activation and the production of IFNs and proinflammatory cytokines (25, 31, 32). HBV polymerase also inhibits IFN-stimulated signal transduction (28, 33). These mechanisms explain how HBV avoids exogenous IFN therapy and maintains chronic infection, and TLR signaling pathways are an essential part of the mechanism. TLRs are evolutionarily well conserved, and 10 members have been identified in humans (33). Some TLRs are localized on endosomes and recognize viral nucleic acids, whereas others are expressed on the cell surface and recognize viral proteins. TLR4 is expressed on the cell surface and is activated by LPS. Activated TLR4 promotes the MyD88-dependent pathway and TAK1 complex activation through TRAF6 E3 ubiquitin ligase. The TAK1 complex then activates the IKK complex and MAPK pathway, which leads to the nuclear translocation of NF-κB and AP-1. NF-κB and AP-1 regulate inflammatory responses by inducing proinflammatory cytokines and IFNs (34). SASH1 functions as a scaffold protein in the TLR4 signaling pathway and promotes the activation of NF-κB by assembling a molecular complex including TRAF6, TAK1, and IKK (18) (Fig. 2F).

In this study, we found that DENND2A interacts with SASH1 and inhibits its role in TLR4 signaling pathway. Considering that DENND2A has the pivotal role in HBV infection, we propose the following function for DENND2A: In hepatocytes infected with HBV, DENND2A traps SASH1 and inhibits NF-κB activation. This leads to the suppression of the innate antiviral immune system, allowing HBV infection to persist (Fig. 2G). To regain the proper operation of the immune system, we identified the novel DENND2A-binding peptide DENP4-3S and incorporate it into the hepatocyte-specific peptide delivery system, which we named 10M-DEN3SN. 10M-DEN3SN effectively inhibits the DENND2A-SASH1 binding and rescued the NF-κB transcriptional activity, resulting in elevated cytokines expression. Of the cytokines whose expression was found to be promoted by 10M-DEN3SN, IFNα and IFNβ have been commonly used in HBV therapy since the 1980s for their strong anti-HBV activity (22). Based on these results, we considered the mechanism of action of 10M-DEN3SN (Fig. 2H). 10M-DEN3SN is endocytosed into hepatocyte (specifically by the anti-ASGR antibody) and releases DENP4-3S inside the cell. In HBV-infected cells, DENP4-3S exhibits the anti-HBV effect by recovering the function of SASH1 restricted by DENND2A. This restoration results in the NF-κB activation in the TLR signaling pathway. On the other hand, DENND2A is known as GEF in the Rab family (6, 7), and SASH1 is reported in tumor suppression, cellular proliferation, and adhesion (35–37). It is necessary to confirm that 10M-DEN3SN primarily interacts with DENND2A and SASH1 *in vivo* and to assess any potential effect on their other functions.

Recently, improved immunomodulators have been investigated for the treatment of HBV. Especially, TLR7 agonist (38–41) and TLR8 agonist (42) are well researched, although their clinical usage is still pending. Our hepatocyte-specific peptide delivery system has already demonstrated inhibitory effects on HBV infection by targeting DOCK11 (4) and DENND2A and offers potential applications to other proteins, including TLR7 and TLR8. The unique aspect of 10M-DEN3SN is its hepatocyte-specific drug delivery in combination with the anti-ASGR antibody and other functional sequences, distinguishing it from siRNA and other inhibitors. This study also suggests a potential synergy when combined with TLR4 agonists or IFNs, as observed with entecavir. 10M-DEN3SN is considered a candidate for combination therapy to eliminate HBV. Furthermore, the system has the potential for further improvement in its effectiveness. We plan to enhance the overall system performance by humanizing the anti-ASGR antibody and optimizing the composition of functional sequences. Simultaneously, we aim to enhance the efficiency of DENND2A-SASH1 inhibition by optimizing the DENP4-3S sequence (e.g., exploring sequences that strongly inhibit the interaction or SASH1 function in the TLR4 signaling pathway).

In conclusion, our research highlights DENND2A and SASH1 in the immune system during HBV infection and 10M-DEN3SN as the potentially effective drug in the treatment of chronic hepatitis B.

## MATERIALS AND METHODS

### Cell culture

Huh7-NTCP-YFP cells were established as previously described (43), and HepG2-NFκB-Luc cells were purchased from Signosis. HEK293T, HepG2, Huh7, Huh7-NTCP-YFP, and HepG2-NFκB-Luc cells were all maintained in DMEM (Nacalai Tesque) supplemented with 10% fetal bovine serum (Sigma) and 1% penicillin and streptomycin in a 5% CO_2_ incubator at 37°C. Additionally, the medium for HepG2 and Huh7 cells contained 2 mM L-glutamine (Nacalai Tesque) and 1 mM sodium pyruvate (Nacalai Tesque), while the medium for Huh7-NTCP-YFP cells included 2 mM L-glutamine, and the medium for HepG2-NFκB-Luc cells is supplemented with 50 µg/mL hygromycin B. For stimulation of TLR4 signaling pathway, LPS (Wako) was used. PXB cells were purchased from PhoenixBio.

### HBV infection

HBV infection and sample treatment of PXB cells (primary human hepatocytes) purchased from PhoenixBio Co. (Hiroshima, Japan) were performed as previously described (3, 4). Briefly, we prepared the culture medium with HBV particles with 5 GEq/cell, 10M-DEN3SN, and entecavir (Tokyo Chemical Industry) at the indicated final concentrations. After 20–28 hours of incubation, the cells were washed once with PBS (Thermo Fisher Scientific) and then cultured with medium containing 2% DMSO and each drug. This medium exchange was performed as indicated, and HBV DNA and cccDNA in the cells were quantified using qPCR.

### Preparation of plasmids

All primer sequences used in this study are listed in Table S2. A DNA clone of DENND2A and SASH1 was purchased from Kazusa DNA Research Institute. The DENND2A gene was cloned into the HisBioFLAG-pcDNA vector to form HisBioFLAG-DENND2A-pcDNA3.3. The mutation in purchased SASH1 cDNA was corrected and then amplified to be cloned into the pcDNA3.3 vector.

The empty vector NLS-GFP-Mock-FH-S28-pcDNA3.3 was constructed by infusion cloning of the functional peptides into the pcDNA3.3 vector and used as N-Mock plasmid. From N-Mock plasmid, N-Ala16 and N-DENAla8 were generated using KOD-Plus-Mutagenesis Kit (Toyobo), and N-DENP4-3 and N-DENP4-3S were generated using In-Fusion Cloning Kit (Clontech).

All plasmid transfections were performed with Lipofectamine 2000 (Invitrogen), and all cell lysis procedures were performed with buffers containing a protease inhibitor cocktail (Nacalai Tesque).

### IVV library construction and screening of DENND2A-binding peptides

IVV selection for DENND2A-binding peptides was performed as previously described (4) with modification of the bait protein. For preparation of the bait protein DENND2A, 293T cells were transfected with HisBioFLAG-DENND2A-pcDNA3.3 for 24 hours. The cells were lysed with lysis buffer (25 mM Tris-HCl pH 7.4, 137 mM NaCl, 2.68 mM KCl, 1% Triton X-100). Lysates were centrifuged at 4°C for 30 min at 13,200 rpm, and the supernatant was applied to Anti-FLAG M2 Agarose Beads (Sigma) at 4°C for 2 hours. HisBioFLAG-DENND2A was eluted with 3× FLAG peptide (Sigma). The eluted proteins were immobilized on the Sensor Chip SA (Cytiva) on a Biacore 3000 system (Cytiva). The IVV library was subjected to the bait-immobilized Sensor Chip SA, followed by photocleavage (44). The recovered RNA was used for the next selection round and sequenced after four rounds of selection. For the anti-HBV assay of the screened peptide, Huh7-NTCP-YFP cells were infected with HBV particles on day 0 and transfected with the plasmid to express the NLS-tagged peptides on day 2. After 10 days of incubation, HBV DNA and cccDNA in the cells were quantified using qPCR.

### Western blotting

HepG2-NFκB-Luc cells were transfected with HisBioFLAG-DENND2A-pcDNA3.3 and then cultured for 24 hours. These cells were lysed with RIPA buffer (Thermo Fisher Scientific) additionally containing a phosphatase inhibitor cocktail (Nacalai Tesque). Lysates were centrifuged at 4°C and 16,000 × *g* for 15 min, and the supernatant was collected. Protein concentrations were determined using a BCA Protein Assay Kit (Thermo Fisher Scientific). Equivalent amounts of protein were separated in NuPAGE 4%–12% Bis-Tris or 3%–8% Tris-Acetate Gel (Thermo Fisher Scientific) or NuPAGE 3%–8% Tris-Acetate Gel (Thermo Fisher Scientific) followed by incubation with primary antibodies against NF-κB, pNF-κB, IKKβ, pIKKα/β, GAPDH (Cell Signaling Technology), DENND2A (SantaCruz), and SASH1 (Novus Biologicals) and with HRP-conjugated antibodies against FLAG-tag (Sigma) and T7-tag (Novagen). HRP-conjugated secondary antibody against rabbit or mouse (Cell Signaling Technology) was then applied. The blots were developed using enhanced chemiluminescence reagents (Cytiva).

### Preparation of 10M-DEN3SN

All amino acid sequences used in the construction of 10M-DEN3SN and 10M-GFP-DEN3SN are listed in Table S1. 10M-DEN3SN was constructed by infusion cloning of the functional peptides into the pHEK293 Ultra Expression Vector (Takara). 10M-GFP-DEN3SN was constructed by infusion cloning of the 10M-DEN3SN and GFP into pcDNA3.3 vector. These plasmids were transfected into HEK293T cells each. These cells were cultured in Expi293 expression medium (Gibco). After 4–6 days, the medium was centrifuged for 10 min at 12,000 × *g,* and the supernatant was collected and applied to a HisTrap HP column (Cytiva). The eluted fraction was applied to Superose6 Increase (Cytiva), and then His-tagged protein was eluted with PBS (Thermo Fisher Scientific). For the preparation of 10M-GFP-DEN3SN, the cells were lysed with RIPA buffer. Lysates were centrifuged at 4°C for 30 min at 16,000 × *g*, and the supernatant was collected and applied to His Mag Sepharose excel (Cytiva) at 4°C overnight. His-tagged protein was eluted with PBS containing 250–500 mM imidazole. The eluted fractions were concentrated using an Amicon Ultra 0.5-mL 10K centrifugal filter (Millipore) followed by buffer exchange to PBS to mix with the purified medium solution. Prepared 10M-GFP-DEN3SN was used to treat Huh7 cells at 8 nM for 5 hours. The cells were lysed and fractionated using an SF-PTS Kit (GL Science). Furin digestion was performed as previously described (4).

### Pulldown assay

All peptides were synthesized by Eurofins genomics. HEK293T cells were transfected with HisBioFLAG-DENND2A or HisT7-SASH1 for 24 hours. These cells were washed with PBS and lysed with RIPA buffer. Lysates were centrifuged at 4°C for 30 min at 16,000 × *g*, and the supernatant was collected. HisBioFLAG-DENND2A in the lysates was immobilized on streptavidin-conjugated magnetic beads (Promega) in TBST. Lysate containing HisT7-SASH1 was mixed with DENND2A-immobilized beads on a rotator for 5 hours at 4°C in the presence of each peptide. The beads were washed with TBST and resuspended in NuPAGE LDS Sample Buffer (1.6×) containing 0.1 M DTT to elute binding proteins.

### Coimmunoprecipitation

HEK293T cells were transfected with HisBioFLAG-DENND2A or HisBioFLAG-vector and HisT7-SASH1 for 48 hours. Cells treated with 0 or 100 nM 10M-DEN3SN for 24 hours were lysed with IP lysis buffer (Thermo Fisher Scientific) containing protease inhibitor cocktail and phosphatase inhibitor cocktail. The lysates were centrifuged at 4°C for 15 min at 16,000 × *g*, and the supernatant was applied to streptavidin-conjugated magnetic beads on a rotator for 3 hours at 4°C. The beads were washed with TBST, and the binding proteins were collected as in a pulldown assay.

### Luciferase assay

HepG2-NFκB-Luc cells were plated at a density of 100,000 cells/well in 24-well plates and grown overnight. The cells were lysed and analyzed for luciferase activity by Luciferase Reporter Assay Kit I (Firefly) (PromoKine) using KC4 plate reader (BioTek Instruments).

### qRT-PCR and ELISA

Cells cultured to 80% confluence were transfected with DENND2A, SASH1, or Universal Negative Control siRNA (Sigma) for 0–72 hours using Lipofectamine 3000 (Invitrogen). qRT-PCR was performed as described previously (4). The primer sets are shown in Table S3. ELISA kit for IFNα (PBL Assay Science) was used according to the manufacturer’s instructions. All samples were incubated overnight at 4°C.

### Immunofluorescence

Huh7 cells on coverslips were treated with 0 or 100 nM 10M-DEN3SN for 5 hours. These cells were then fixed with 10% paraformaldehyde for 10 min followed by permeabilization with 0.2% Triton X-100 (Nacalai Tesque) for 10 min. The samples were stained with antibodies against DENND2A, SASH1 (Abnova), and NF-κB, PE-conjugated FLAG-tag (Cell Signaling Technology), and FLAG-tag followed by Alexa488 or Alexa647-conjugated anti-rabbit or mouse IgG (Invitrogen). DAPI solution (Thermo Fisher Scientific) was used for nuclear staining. Photobleaching was prevented using Slow Fade Gold antifade reagent (Invitrogen). The cells were observed with an LSM800 confocal microscope (Zeiss) and analyzed using ZEN software (Zeiss). The fluorescence intensity of the target protein was normalized to that of DAPI.

### Mice

All experiments performed in mice were approved by AAALAC International and performed as previously described (4). Briefly, SCID mice were constructed by PhoenixBio Co. Ltd. Among the cDNA-uPAwild/+/SCID mice transfected with human hepatocytes, we used PXB chimeric mice with an expected replacement rate of human hepatocytes greater than 70%, calculated based on human albumin levels in mouse blood. Groups of PXB chimeric mice (5/group) were dosed with 10M-DEN3SN or PBS via intraperitoneal administration at 300 µL/mouse once every 2–3 days from the eighth week of virus inoculation (day 0) to the day before specimen collection (day 27). Blood samples were collected at week 6 (2 weeks before the first dose) and once a week from before the first dose to day 21, and at necropsy (day 28), all available blood samples were collected. The concentration of h-Alb in blood and concentration of HBeAg, HBsAg, HBV DNA, and ALT in serum were measured. HBV DNA and cccDNA in the livers were measured on day 28. The sequences of the primers and probes are shown in Table S4.

In addition, liver tissues were fixed in 10% neutral buffered formalin solution and replaced with 70% ethanol. These samples were paraffin embedded at the Nara Institute of Pathology, and thin section specimens were subsequently obtained. The paraffin sections were deparaffinized, and antigen activation was performed by microwaving. Primary antibodies against HBsAg (Bio-Rad) or HBcAg (Abnova) were reacted at 4°C overnight. The primary antibodies were reacted with the biotin-avidin-peroxidase complex and then colorized by DAB. After staining the cell nuclei with hematoxylin, these sections were dehydrated, permeabilized, and sealed. Subsequently, a mirror examination was performed using an optical microscope at Hamri Co.

### Statistical analysis

The data were obtained from three or more independent experiments. For all data, comparisons were made using Student’s *t*-test. All data are shown as the means ± SD with *P* < 0.05 considered statistically significant. *P < 0.05; **P < 0.005; n.s., not significant.

## Data Availability

All correspondence and material requests should be addressed to the corresponding author, H.Y.

## SUPPLEMENTAL MATERIAL

Fig. S1 to S7, Tables S1 to S5 spectrum.03785-23-s0001.pdf Supplemental figures and tables.

ASM does not own the copyrights to Supplemental Material that may be linked to, or accessed through, an article. The authors have granted ASM a non-exclusive, world-wide license to publish the Supplemental Material files. Please contact the corresponding author directly for reuse.
