# Supplementary material for: Hepatitis B virus evades the immune system by suppressing the NF-κB signaling pathway with DENND2A
Source: Microbiol Spectr. 2024 Jan 19;12(3):e03785-23. doi: 10.1128/spectrum.03785-23 (PMC10913737; doi:10.1128/spectrum.03785-23)
Supplement: Fig. S1 to S7, Tables S1 to S5 — Supplemental figures and tables. [file spectrum.03785-23-s0001.pdf]

Fig S1.

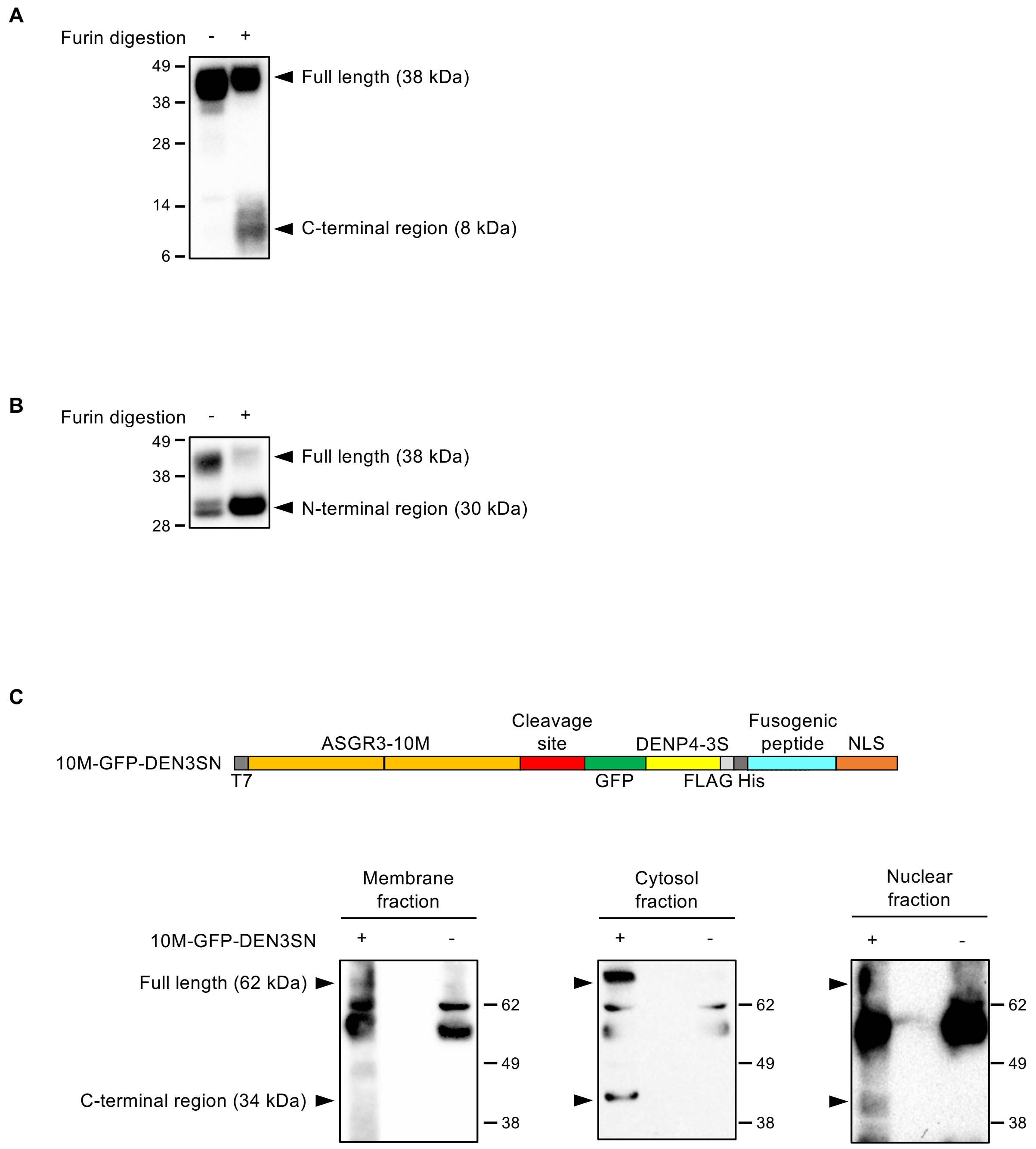

Fig S1

(**A, B**) 10M-DEN3SN was treated with the protease Furin at 37 ° C for 1 hour, separated in 4-12% SDS–PAGE and analyzed by western blotting with antibodies against FLAG-tag (**A**) and T7-tag (**B**).

(**C**) Huh7 cells were treated with 10M-GFP-DEN3SN (10M-DEN3SN specially containing GFP) for 5 hours. The cells were lysed and fractionated to membrane, cytosol or nuclear fraction. Each fraction was separated by 4-12% SDS–PAGE and then analyzed by western blotting with an antibody against the FLAG-tag.

Fig S2

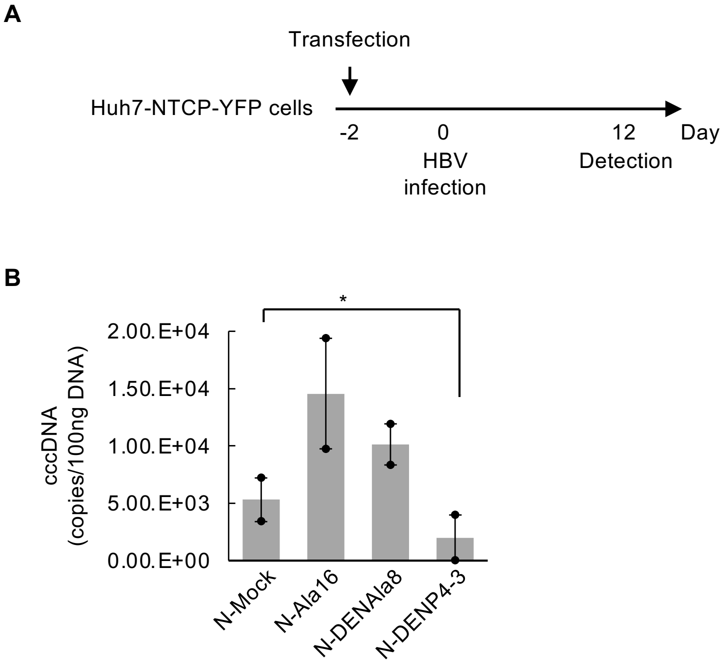

Fig S2

Anti-HBV effect of peptides used as negative controls. (**A**) Schematic of the experimental design. (**B**) Huh7-NTCP-YFP cells were transfected with a plasmid encoding NLS-tagged Ala16 (N-Ala16), DENAla8 (N-DENAla8), DENP4-3 (N-DENP4-3) and then infected with HBV for 12 days. cccDNA levels were measured using qPCR. Data are presented as the mean ± SD pooled from two independent experiments. *p < 0.05.

Fig S3

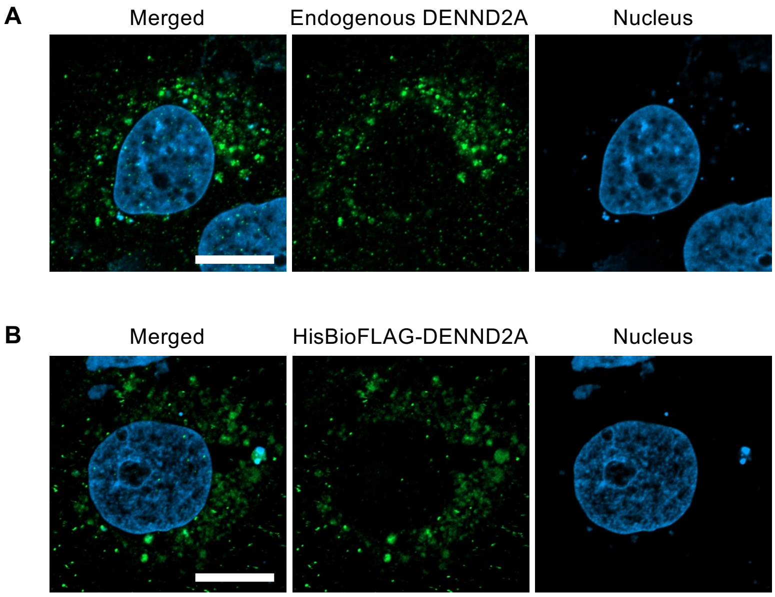

Fig S3

Localization of endogenous or overexpressed DENND2A. (**A**) Immunofluorescence staining of Huh7 cells using anti-DENND2A antibody (Alexa488, green). DAPI was used to stain nuclei (blue). (**B**) Immunofluorescence staining of Huh7 cells using anti-FLAG-tag antibody (Alexa488, green) after transfection with HisBioFLAG-DENND2A for 24 h. The samples were observed under a fluorescence microscope. Scale bars, 10 μm.

Fig S4

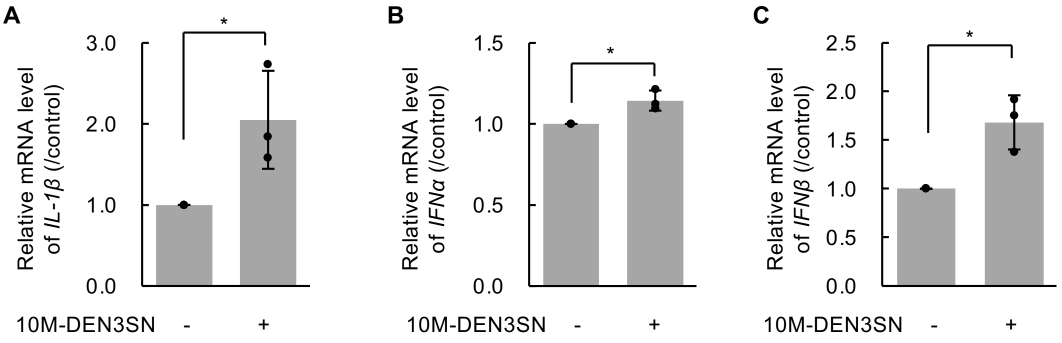

Fig S4

10M-DEN3SN promoted the NF-κB signaling pathway in HepG2-NFκB-Luc cells. HepG2-NFκB-Luc cells were treated with 100 nM 10M-DEN3SN for 5 h, and *IL-1β* (**A**), *IFNα* (**B**) and *IFNβ* (**C**) mRNA were detected by qRT–PCR. Error bars represent ±SD. *p < 0.05.

Fig S5

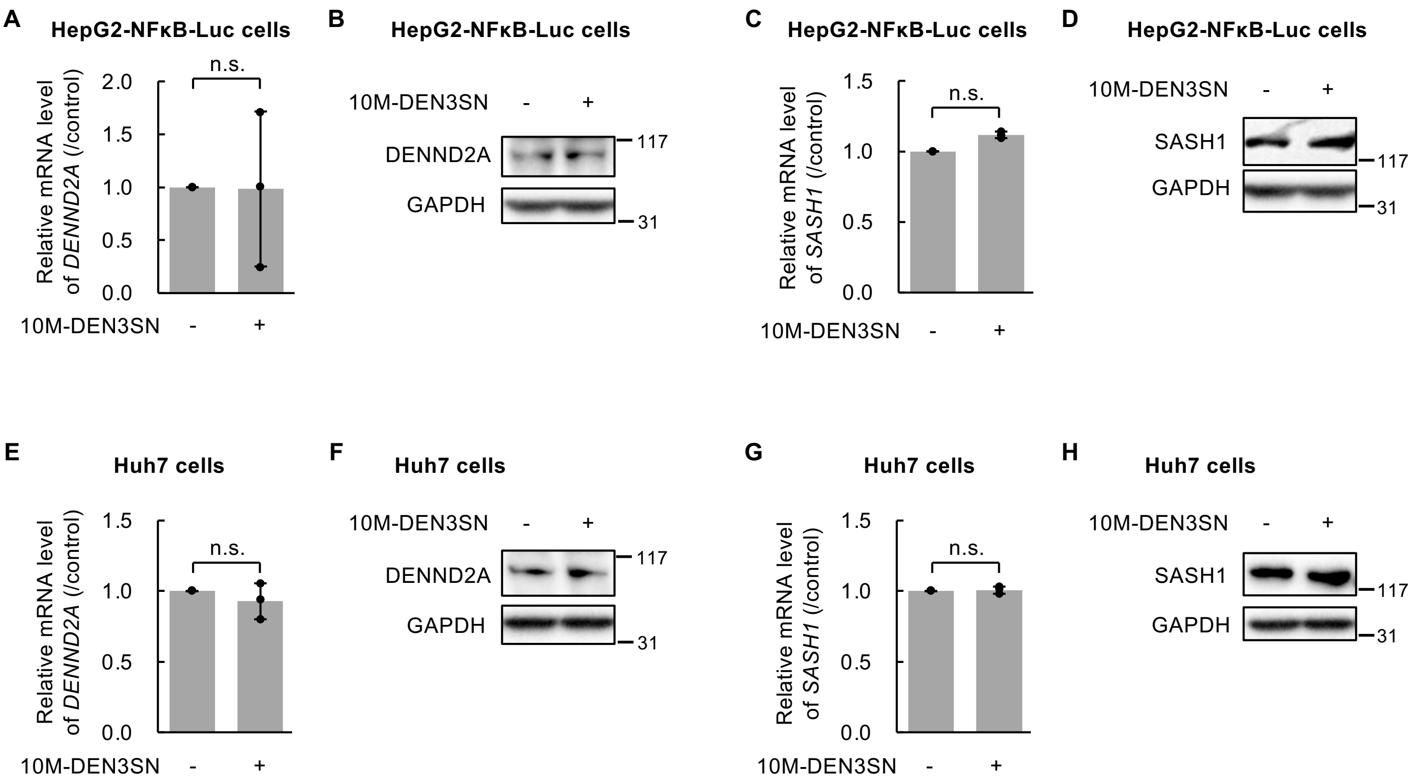

Fig S5 10M-DEN3SN had no effect to the mRNA or protein level of DENND2A and SASH1.

(**A-D**) HepG2-NFκB-Luc cells were treated with 0 or 100 nM 10M-DEN3SN for 5 h. DENND2A or SASH1 mRNA was detected by qRT–PCR (**A, C**), and their protein levels were detected by western blotting (**B, D**). GAPDH is shown to verify equal loading.

(**E-H**) Huh7 cells were treated with 0 or 100 nM 10M-DEN3SN for 5 h. DENND2A or SASH1 mRNA was detected by qRT–PCR (**E, G**), and their protein levels were detected by western blotting (**F, H**). GAPDH is shown to verify equal loading.

Error bars represent ±SD. n.s.: not significant.

Fig S6

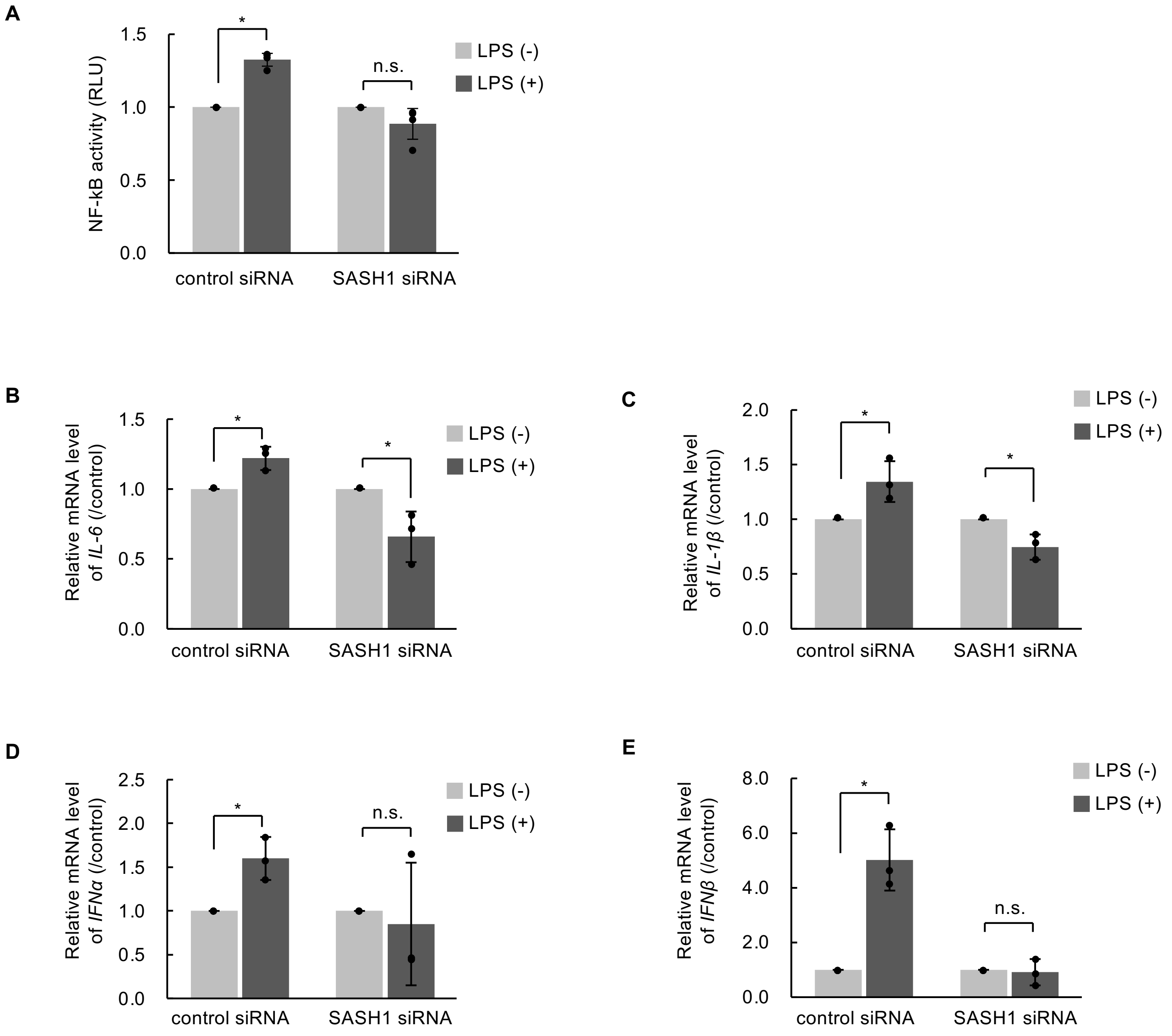

Fig S6

SASH1 promoted NF-κB signaling pathway through TLR4 signaling pathway. (**A**) HepG2-NFκB-Luc cells were transfected with SASH1 or control siRNA for 72 h and then treated with 0 or 250 ng/ml LPS for 5 h. The NF-κB transcriptional activity was measured by luciferase assay. (**B-E**) Huh7 cells were transfected with SASH1 or control siRNA for 72 h and then treated with 0 or 250 ng/ml LPS for 5 h. IL-6 (**B**), IL-1β (**C**), IFNα (**D**) and IFNβ (**E**) mRNA were detected by qRT–PCR. Error bars represent ±SD. *$p < 0.05$; n.s.: not significant.

Fig S7

A

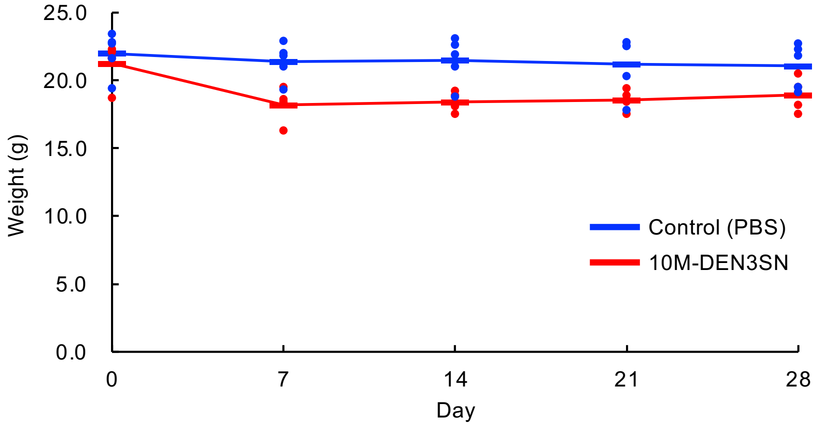

B

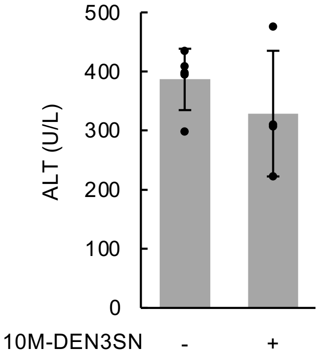

Fig S7

10M-DEN3SN causes no toxicity in PXB chimeric mice. (**A**) The body weight of mice for 0-28 days. (**B**) ALT in the serum at necropsy on Day 28.

Table S1 Amino acid sequences of 10M-DEN3SN and functional peptides used in the construction of 10M-DEN3SN.

Amino acid sequences of 10M-DEN3SN.

Name	Sequence (5' to 3')
10M-DEN3SN	MASMTGGQQMGQVQLQQSGAELMKPGASVKISCKATGYTFSSYWIEWVKQRPG<br>HGLEWIGEILPGSGSTNYNEKFKGKATFTADTSSNTAYMQLSSLTSEDSAVYYCAR<br>YGNYDAMDYWGQGTTLTVSSSGGGGSGGGGSGGGGSGGGGSDIVLTQSPATLS<br>LSPGERATLSCRASQSVSSSYLAWYQQKPGQAPRLLIYYASSRATGVPARFSGSG<br>SGTDFTLTISSLEPEDFAVYYCQQSSSTPFTFGQGTKVEIKRRVRRLPSPDAPCLP<br>VKRGSPDYKDDDDKHHHHHHHPFVIGAGVLGALGTGIGGITTSTQFYYKPAAKRV<br>KLD

Amino acid sequences of functional peptides used in the construction of 10M-DEN3SN and 10M-GFP-DEN3SN.

Name	Sequence (5' to 3')
T7-tag	MASMTGGQQMG
ASGR3-10M	QVQLQQSGAELMKPGASVKISCKATGYTFSSYWIEWVKQRPGHGLEWIGEILPGS<br>GSTNYNEKFKGKATFTADTSSNTAYMQLSSLTSEDSAVYYCARYGNYDAMDYWG<br>QGTTLTVSSSGGGGSGGGGSGGGGSGGGGSDIVLTQSPATLSLSPGERATLSCR<br>ASQSVSSSYLAWYQQKPGQAPRLLIYYASSRATGVPARFSGSGSGTDFTLTISSLE<br>PEDFAVYYCQQSSSTPFTFGQGTKVEIKR
cleavage site	RVRR
GFP	SKGEELFTGVVPILVELDGDVNGHKFSVSGEGEGDATYGKLTLKFICTTGKLPVPW<br>PTLVTTLCYGVQCFSRYPDHMKRHDFFKSAMPEGYVQERTIFFKDDGNYKTRAEV<br>KFEGDTLVNRIELKGIDFKEDGNILGHKLEYNYNSHNVYIMADKQKNGIKVNFKTR<br>HNIEDGSVQLADHYQQNTPIGDGPVLLPDNHYLSTQSALSKDPNEKRDHMVLLEF<br>VTAAGITHGMDELYK
FLAG-tag	DYKDDDDK
His-tag	HHHHHHH
Fusogenic peptide S28	PFVIGAGVLGALGTGIGGITTSTQFYYK
Nuclear Localization Sequence (NLS)	PAAKRVKLD

Table S2 Primers for DENND2A, SASH1, ASGR and peptides expression vectors and for mRNA selection.

Primers for DENND2A expression vector

Name	Sequence (5' to 3')
F-Bio	GGCGCCGGCACCCCGGTGAC
R-Bio	CGCCAGGGTCATCAGGGTGT
KstartHisBio-F	CGGACTCTAGAGGATGCCACCATGCACCATCACCACCATCACCATGGCGCCGGCACCCCGGTGAC
BioFLAGstop-INV-R	CTAACCGGTAGGGATCTACTTGTCGTCGTCGTCCTTGTAGTCCGCCAGGGTCATCAGGGTG
DENND2A-ifFLAG-F	GACGACGACGACAAGATGGATATGTTCAGCTTGGA
DENND2Ai2-ifpc-R	CTAACCGGTAGGGATCTAGAGGGTCCAAGAGCGAC
Stoptopo-INV-F	TAGATCCCTACCGGTTAGTAATGAGTTTAAACG
FLAG-R	CTTGTCGTCGTCGTCCTTGTAGTC

Primers for SASH1 expression vector

Name	Sequence (5' to 3')
SASH1mut-F	TATCAGGGACCCGGAACACAGAGCTG
SASH1mut-R	TTTAACTCATCCAAGTCTTCCTCCTCCAGC
HisT7-SASH1-f	ATGCACCATCACCACCATCACCATATGGCTAGCATGACTGGTGGACAGCAAATGGGTATGGAGGACGCGGGAGCAGCTGGCC
SASH1-R	CTACATGGCCTCAGGGCCTGGCGGC

Primers for ASGR expression vector

Name	Sequence (5' to 3')
ASGR1-ex-if-F1	GACGACGACGACAAGCAAAACTCCCAGCTGCAGGA
ASGR2-ex-if-F1	GACGACGACGACAAGCAAAGTGAGGGTCACAGGGG
ASGR1-if-R2	CTAACCGGTAGGGATTTAAAGGAGAGGTGGCTCCT
ASGR2-if-R2	CTAACCGGTAGGGATTCAGGCCACCTCGCCGGTGG

Primers for NLS-tagged peptides expression vectors

Peptide	Name	Sequence (5' to 3')
Ala16	FlagHis-F	GACTATAAGGACGACGACGACAAGCACCATCACC
	GFP-ALA16R	GGCAGCTGCGGCAGCTGCTGCGGCAGCTGCGGCAGCTGCGGCAGCTGCTTTGTATAGTTCATCCATGCC
DENala8	PFlagHis-F	CCCGACTATAAGGACGACGACGACAAGCACCATCAC
	DEN-Ala8-R	CGCAGCTGCAGCTGCAGCTGCAGCTGGCGCATCGGGACTGGGGAG
DENP4-3	DENP4-3-F	GATGAACTATACAAAATCCACGACCGGTGCACCGGCTGCCTCCCCGTCAAGC
	DENP4-3-R	GTCGTCCTTATAGTCGTGGGACCCCCGCTTGACGGGGAGGCAGCCGGTGC
DENP4-3S	DENP4-3SA-F	GATGAACTATACAAACTCCCCAGTCCCGATGCGCCATGCCTGCCAGTGAAAAG
	DENP4-3SA-R	GTCGTCCTTATAGTCGGGGCTGCCCCTTTTCACTGGCAGGCATGGCGCATC

Primers for mRNA selection

Name	Sequence (5' to 3')
GSP6-GFP-F	GGAAGATCTATTTAGGTGACACTATAGAACAACAACAACAACAAACAACAACAAAATGAGTAAAGGAGAAGAACTTTTCACTGGAG
GFP-R	TTTGTATAGTTCATCCATGCCATGTG
Flag-His-F	GACTATAAGGACGACGACGACAAGCACCATC
Atail (R)	TTTTTTTTTCTTGTAGTAGAACTGGGTGCTGGTCGTAATCCCGCCAATGCC
9NNS-F	CATGGCATGGATGAACTATACAAANNSNNSNNSNNSNNSNNSNNSNNSNNSGACTATAAGGACGACGACG
16NNS-F	GGATGAACTATACAAANNSNNSNNSNNSNNSNNSNNSNNSNNSNNSNNSNNSNNSNNSNNSNNSGACTATAAGGACGACGACG

Table S3 Primers for qRT–PCR targeting DENND2A, SASH1, IL-6, IL-1β, IFNα, IFNβ and GAPDH.

Primers for qRT-PCR

Name	Sequence (5' to 3')
DENND2A Forward primer	AACTGAAGGCCATTCCCCAG
DENND2A Reverse primer	TCTTCGGCAGTAACCGAACC
SASH1 Forward primer	CCCACTTTCCTGTTCAATG
SASH1 Reverse primer	TGGTCGCTGTTACTGTCATAC
IL-6 Forward primer	ACAGCCACTCACCTCTTCAG
IL-6 Reverse primer	CCATCTTTTTCAGCCATCTTT
IL-1β Forward primer	AGATGATAAGCCCACTCTACAG
IL-1β Reverse primer	ACATTCAGCACAGGACTCTC
IFNα1 Forward primer	TAGACAAATTCTGCACCGAAC
IFNα1 Reverse primer	AGATGGAGTCCGCATTCATC
IFNβ1 Forward primer	TTGACATCCCTGAGGAGATTAAGC
IFNβ1 Reverse primer	TTAGCCAGGAGGTTCTCAACAATAG
GAPDH Forward primer	GGAAGGACTCATGACCACA
GAPDH Reverse primer	GGAAGGACTCATGACCACA

Table S4 Primers for PCR to assess HBV DNA and cccDNA levels.

Primers for PCR to assess HBV DNA levels

Name	Sequence (5' to 3')
Forward primer	CACATCAGGATTCCTAGGACC
Reverse primer	AGGTTGGTGAGTGATTGGAG
TaqMan probe	6-FAM-CAGAGTCTAGACTCGTGGTGGACTTC-TAMRA

Primers for PCR to assess cccDNA levels

Name	Sequence (5' to 3')
Forward primer	CTCCCCGTCTGTGCCTTCT
Reverse primer	GCCCCAAAGCCACCCAAG
TaqMan probe	6-FAM-CGTCGCATGGARACCACCGTGAACGCC-TAMRA

Table S5 The statistical values for Fig. 6 and Fig. S7.

Group	Mouse	Weight（g） Day 0	7	14	21	28	%/Day 0
10M-DEN3SN	1	18.7	16.3	17.5	17.5	17.5	(94%)
	2	22.3	18.4	19.2	19.4	20.5	(92%)
	3	21.6	19.5	18.1	18.4	18.2	(84%)
	4	21.6	18.6	18.9	18.9	19.5	(90%)
	5	22.1					
	Mean	21.3	18.2	18.4	18.6	18.9	
	SD	1.5	1.4	0.8	0.8	1.3	
PBS	1	19.4	19.3	18.8	17.8	19.1	(98%)
	2	22.7	21.8	21.9	22.5	21.8	(96%)
	3	22.8	22.0	22.6	22.5	22.3	(98%)
	4	21.6	21.0	21.0	20.3	19.5	(90%)
	5	23.4	22.9	23.1	22.8	22.7	(97%)
	Mean	22.0	21.4	21.5	21.2	21.1	
	SD	1.6	1.4	1.7	2.1	1.7	

		HBeAg (C.U.I)					HBsAg (C.U.I)					HBV DNA in serum（copies/mL）					ALT (U/L)	HBV DNA (copies/100ng DNA)	cccDNA (copies/100ng DNA)
Group	Mouse	Day					Day					Day					Day	Day	Day
		0	7	14	21	28	0	7	14	21	28	0	7	14	21	28	28	28	28
10M-DEN3SN	1	228	996	2130	1248	834	194	648	2172	948	696	4.0.E+06	5.6.E+07	4.0.E+07	8.3.E+07	7.6.E+07	222	2.0.E+05	9.1.E+03
	2	762	2910	1962	2346	1776	557	2076	1452	2052	2094	1.9.E+07	4.1.E+08	7.2.E+07	1.4.E+08	1.4.E+08	307	3.2.E+05	1.3.E+04
	3	480	1338	1530	1698	1314	360	1158	1848	1944	1560	4.7.E+07	1.1.E+08	2.4.E+08	1.9.E+08	7.7.E+07	310	2.4.E+05	1.2.E+04
	4	84	354	612	1050	1764	39	210	443	870	1782	2.8.E+06	5.3.E+06	4.2.E+07	3.2.E+07	2.8.E+06	476	4.0.E+04	1.7.E+03
	5	2262					2130					5.5.E+08							
	Mean	763.2	1399.5	1558.5	1585.5	1422.0	656.1	1023.0	1478.7	1453.5	1533.0	1.3.E+08	1.5.E+08	1.0.E+08	1.1.E+08	7.3.E+07	329	2.0.E+05	8.9.E+03
	SD	876.7	1086.5	679.7	574.9	447.1	846.2	801.8	750.7	631.1	599.4	2.4.E+08	1.8.E+08	9.8.E+07	6.7.E+07	5.5.E+07	106	1.2.E+05	5.1.E+03
PBS	1	2346	3588	3174	3984	3120	2682	4128	3894	4578	3594	5.7.E+08	2.2.E+08	7.4.E+08	3.2.E+08	3.3.E+08	398	5.3.E+05	3.8.E+04
	2	774	2508	2472	2292	2940	371	1596	2538	2088	2652	6.6.E+07	2.9.E+08	2.9.E+08	1.7.E+08	2.3.E+08	434	3.6.E+05	1.6.E+04
	3	1290	2256	2340	2688	1644	822	1812	2094	2688	2004	1.4.E+08	2.7.E+08	2.1.E+08	1.9.E+08	9.3.E+07	409	2.3.E+05	9.8.E+03
	4	750	1668	1884	2058	1668	420	1302	2004	1842	1818	8.4.E+06	4.8.E+07	7.0.E+07	1.1.E+08	1.0.E+08	298	2.2.E+05	1.2.E+04
	5	348	1452	2166	2682	2520	202	870	1590	2178	2232	1.8.E+07	7.5.E+07	3.3.E+08	1.8.E+08	3.2.E+08	394	3.8.E+05	1.5.E+04
	Mean	1101.6	2294.4	2407.2	2740.8	2378.4	899.5	1941.6	2424.0	2674.8	2460.0	1.6.E+08	1.8.E+08	3.3.E+08	1.9.E+08	2.2.E+08	387	3.4.E+05	1.8.E+04
	SD	771.8	840.0	481.8	744.9	694.5	1022.1	1272.2	888.1	1107.6	706.2	2.3.E+08	1.1.E+08	2.5.E+08	7.6.E+07	1.2.E+08	52	1.3.E+05	1.1.E+04
